# Immune evasion by *Cryptococcus gattii* in vaccinated mice coinfected with *C. neoformans*

**DOI:** 10.3389/fimmu.2024.1356651

**Published:** 2024-02-26

**Authors:** Maureen M. Hester, Diana Carlson, Jennifer K. Lodge, Stuart M. Levitz, Charles A. Specht

**Affiliations:** ^1^ Department of Medicine, The University of Massachusetts Chan Medical School, Worcester, MA, United States; ^2^ Department of Molecular Genetics and Microbiology, Duke University School of Medicine, Durham, NC, United States

**Keywords:** *Cryptococcus*, fungal vaccine, coinfection, KN99, R265, immune evasion, immune suppression

## Abstract

*Cryptococcus neoformans* and *C. gattii*, the etiologic agents of cryptococcosis, cause over 100,000 deaths worldwide every year, yet no cryptococcal vaccine has progressed to clinical trials. In preclinical studies, mice vaccinated with an attenuated strain of *C. neoformans* deleted of three cryptococcal chitin deacetylases (*Cn*-*cda1*Δ*2*Δ*3*Δ) were protected against a lethal challenge with *C. neoformans* strain KN99. While *Cn-cda1*Δ*2*Δ*3*Δ extended the survival of mice infected with *C. gattii* strain R265 compared to unvaccinated groups, we were unable to demonstrate fungal clearance as robust as that seen following KN99 challenge. In stark contrast to vaccinated mice challenged with KN99, we also found that R265-challenged mice failed to induce the production of protection-associated cytokines and chemokines in the lungs. To investigate deficiencies in the vaccine response to R265 infection, we developed a KN99-R265 coinfection model. In unvaccinated mice, the strains behaved in a manner which mirrored single infections, wherein only KN99 disseminated to the brain and spleen. We expanded the coinfection model to *Cn-cda1*Δ*2*Δ*3*Δ-vaccinated mice. Fungal burden, cytokine production, and immune cell infiltration in the lungs of vaccinated, coinfected mice were indicative of immune evasion by *C. gattii* R265 as the presence of R265 neither compromised the immunophenotype established in response to KN99 nor inhibited clearance of KN99. Collectively, these data indicate that R265 does not dampen a protective vaccine response, but rather suggest that R265 remains largely undetected by the immune system.

## 1 Introduction

*Cryptococcus neoformans* and *C. gattii* are the main etiologic agents of cryptococcosis, a fungal infection occurring predominately in immunocompromised populations. Both species can be found in living trees and decaying wood, with one niche of *C. neoformans* uniquely being pigeon guano. Inhalation of *Cryptococcus* and subsequent colonization of the lungs can cause pneumonia-like symptoms. However, pulmonary disease often goes undiagnosed until dissemination to the central nervous system and the onset of cryptococcal meningitis (CM). CM causes over 100,000 deaths annually and is responsible for approximately 19% of AIDS-related mortalities (1). Most of these cases are associated with the opportunistic pathogen *C. neoformans*, which primarily afflicts immunodeficient individuals. In addition to persons with AIDS, other vulnerable groups include those regularly taking immunosuppressive medications, such as recipients of solid organ transplants. While exposure is predicted to be common due to its broad global distribution, persons with intact immune systems generally are able to eliminate the yeast (2). Contrastingly, *C. gattii* more readily infects the immunocompetent population, and disease tends to persist as a pulmonary rather than a systemic infection (3–5). Historically these infections are much rarer than those due to *C. neoformans* and have been seen across a comparatively restricted geographic range (5). Over the past several decades, however, *C. gattii*, previously considered to be confined to tropical and subtropical climates, became endemic in British Columbia, Canada, and the Pacific Northwest of the United States (5, 6). The resulting increase in infections highlights the need for vaccine development.

While no cryptococcal vaccines have proceeded to clinical trial, there has been significant progress in the development of experimental vaccine formulations which protect mice against infection. Many of these have been tested against hypervirulent strains of *C. neoformans* and *C. gattii*. One prominent laboratory strain of *C. neoformans* is KN99α. KN99 is an isogenic derivative of H99, a *C. neoformans* var. *grubii* reference strain originally isolated from a patient with CM (7). *C. gattii* strain R265 is an isolate obtained from a patient during the outbreak in British Columbia (8). R265 is molecular type VGIIa, the molecular type most often found to cause disease in both humans and animals (9).

Over 45 different anti-cryptococcal vaccines have been tested in mouse models [reviewed in (10) and (11)]. One common immunization strategy is the use of whole organism vaccines derived from cryptococcal mutants, and various approaches have been taken to generate these attenuated strains. One approach was engineering *Cryptococcus* to express particular gene products. A strain that overexpresses the Znf2 transcription factor, which regulates the yeast to hyphae transition is protective even in a heat-killed form (12); while H99γ produces murine IFNγ and promotes a T helper (Th) 1 response upon subsequent challenge (13). Other groups have focused on cryptococcal deletion strains for vaccination. Elimination of F-box protein (Fbp1), sterylglucosidase (Sgl1), or plasma membrane proteolipid 3-related membrane protein 1 (Prm1) expression generated strains *fbp1*Δ*, sgl1*Δ, and *prm1*Δ, respectively (14–16). While the precise mechanism of protection for these strains remains unclear, each induced a Th1 response following vaccination (14–16). We have also demonstrated the efficacy of an avirulent, cryptococcal mutant strain: Deletion of all three cryptococcal chitin deacetylase (Cda) genes yielded a chitosan-deficient strain of *C. neoformans*, *cda1*Δ*2*Δ*3*Δ (*Cn-cda1*Δ*2*Δ*3*Δ) (17). *Cn-cda1*Δ*2*Δ*3*Δ was rapidly cleared from the lungs following inoculation and selectively enriched for protective Th1 populations (17).

Despite the efficacy of experimental vaccines at protecting mice against *C. neoformans* H99 and KN99 infection, generating substantial immunity to *C. gattii* R265 infection has presented researchers with more difficulties. *C. neoformans* and *C. gattii* share up to 90% genomic identity (18), but distinct differences between H99 and R265 have been documented. The impact of these strain-specific phenotypes on virulence and vaccine response, however, is still unclear. Multiple groups have shown the ability of their vaccines to extend survival of R265-challenged mice compared to unvaccinated controls; however, surviving animals in these studies often retain a high fungal burden in their lungs (13, 14, 17, 19–23). We have also been unable to promote significant clearance of R265 from the lungs of vaccinated mice, regardless of whether the vaccine strain was *C. neoformans*-derived *Cn-cda1*Δ*2*Δ*3*Δ or derived from *C. gattii* (*Cg-cda1*Δ*2*Δ*3*Δ) (17, 22).

The studies presented here aimed to identify deficiencies in *Cn-cda1*Δ*2*Δ*3*Δ-mediated protection following challenge with *C. gattii* R265 and to elucidate the driving forces behind these deficiencies. We first established that our *in vivo* challenge model aligned with previously published phenotypes for *C. neoformans* and *C. gattii* infections in a murine model. We then investigated cytokine and chemokine responses in the lungs of *Cn-cda1*Δ*2*Δ*3*Δ-vaccinated mice. Disparate inflammatory signatures comparing the two strains raised the question of whether this was due to R265 suppression of the immune response or its ability to go undetected in the lungs. To address this, we coinfected mice with KN99 and R265, which served as a unique tool to gauge strain-dependent immunological phenomena. We used this approach to compare strain-specific fungal clearance, cytokine production, and leukocyte infiltration in the lungs of unvaccinated and *Cn-cda1*Δ*2*Δ*3*Δ-vaccinated mice. Our findings suggest that R265 is evading immune detection rather than suppressing the induction of an otherwise protective response.

## 2 Materials and methods

### 2.1 Chemicals and culture media

Reagents were from Thermo Fisher Scientific (Pittsburgh, PA) unless otherwise stated. Culture media for *Cryptococcus* was YPD (Difco yeast extract, Bacto peptone, dextrose, with and without 2% agar), and Sabouraud dextrose agar (Remel). Antibiotic selection was with Geneticin (G418) (200 μg/mL, Gibco).

### 2.2 Strains of *Cryptococcus*

*C. neoformans* var. *grubii* strains KN99 (7, 24), KN99mCH (JLCN 920) (25), and *Cn-cda1*Δ*2*Δ*3*Δ (26) all generated on the H99 background; *C. gattii* strain R265 (27); and *Cg-cda1*Δ*2*Δ*3*Δ (22), generated on the R265 background, were maintained as glycerol stocks at -80°C. Initial cultures were grown on YPD agar medium and served as stock plates to inoculate liquid YPD medium. The *cda1*Δ*2*Δ*3*Δ strains were cultured for orotracheal (OT) vaccination in 20 mL of YPD using 250 mL vented flasks with shaking for 2 days at 225 rpm at 30°C. Cultures for subcutaneous (SQ) vaccination were prepared in 4 mL of YPD using 14 mL culture tubes angled at 70 degrees and incubated under the same conditions. The challenge strains (KN99, KN99mCH, and R265) were cultured in 4 mL liquid YPD at 30°C with shaking for ~18 hrs. Cells were harvested by centrifugation at 425xg for 5 min and washed twice with 4 mL (4 mL cultures) or 10 mL (20 mL cultures) of PBS. Cell counts were obtained using a TC20 automated cell counter (BioRad, Hercules, CA). Concentrations were adjusted to 2x10^8^, 2x10^7^, 4x10^5^, or 2x10^5^ cells/mL in PBS for OT vaccination, SQ vaccination, or challenge, respectively. CFU of the inoculum was verified by plating on Sabouraud dextrose agar. Cell counts and colony forming units (CFU) are normally equivalent.

### 2.3 Mice

BALB/c mice (strain #000651) of both sexes were purchased from The Jackson Laboratory (Bar Harbor, ME). Mice were housed and bred in a pathogen-free environment at the University of Massachusetts Chan Medical School (UMCMS). All experiments were approved by the UMCMS School Institutional Use and Care of Animals Committee.

### 2.4 Vaccination and challenge

Mice received a series of three *cda1*Δ*2*Δ*3*Δ vaccinations, with two weeks between each. The first in the series was administered orotracheally: mice were anesthetized with isoflurane, and 1x10^7^ cells in 50 μL of PBS were inoculated into the lung. This was followed by two subcutaneous boosts of 2x10^6^ cells in 100 μL of PBS, as previously described (17). Two weeks following the final vaccination, mice were challenged by OT infusion into the lung. For our standard challenge model, mice were infected with 2x10^4^ cells of the indicated strain in 50 μL of PBS. KN99mCH was used for coinfection studies. For the coinfection model, singly infected mice received 1x10^4^ cells of the indicated strain. Coinfected mice received 2x10^4^ cells total, which was composed of 1x10^4^ cells of KN99mCH and 1x10^4^ cells of R265. Mice were either euthanized at the indicated time points, or survival was monitored daily until 70 days post infection (DPI) before survivors were euthanized. For some experiments, mouse lungs were separated for multiple analyses. In these cases, the lungs were divided such that the apical, azygous, diaphragmatic, and cardiac lobes of the lung (representing 66% of total lung, designated “right lung”) were processed together, and the left lobe of the lung (representing 34% of total lung, designated “left lung”) was processed separately. Based on relative weight of the lung lobes, a factor of 1.515 and 2.941 was used for calculating lung totals based off of right and left lung results, respectively.

### 2.5 Determination of fungal burden

Mice were euthanized via CO_2_ asphyxiation, and lungs, brains, and spleens were collected for analysis. Dissected lungs were homogenized in 4 mL of PBS with 200 U/mL penicillin and 200 μg/mL streptomycin (PBS PenStrep), and brains and spleens were homogenized in 2 mL of PBS PenStrep. Homogenization was done using an OMNI TH tissue homogenizer with tip adaptor for 7 mm x 110 mm hard tissue probe (OMNI International, Kennesaw, GA). Homogenates were plated on Sabouraud dextrose agar and incubated at 30°C for two days before CFUs were counted. Tissue homogenates from coinfected mice were plated on Sabouraud dextrose agar and also YPD agar medium containing 200 μg/mL G418 to select for KN99mCH CFU, which is G418 resistant (25).

### 2.6 Phenol sulfuric assay

Carbohydrate content in the lung was determined by the phenol sulfuric assay (28). Uninfected, 7, and 14 DPI lung samples were homogenized in 4 mL of PBS PenStrep, while 21 DPI lungs were processed in 10 mL. Aliquots from lung homogenates were centrifuged at 20,800xg for 5 min, and the supernatants were collected for analysis. Samples were diluted 1:40 in water for the assay (10 μL of sample plus 390 μL of water, before adding 10 μL of phenol). Buffer-saturated phenol (Invitrogen) was mixed with samples before the rapid addition of 1 mL concentrated sulfuric acid. Aliquots of 100 μL were transferred to a 96-well plate and absorbance was read at 485 nm. Glucose at 0.5-10 mg/mL was used to generate standard curves.

### 2.7 Microscopy

Lungs were dissected and processed into a single cell suspension using the MACS Lung Dissociation Kit per the manufacturer’s recommendations (Miltenyi Biotec, Waltham, MA). Briefly, lungs were subjected to enzymatic digestion combined with mechanical dissociation. Following dissociation, cells were washed twice in 10 mL of PBS. The final suspension volume was adjusted empirically based on cell density and ranged from 2 mL to 10 mL. For microscopy, cell suspensions were mixed with India ink [2-3 parts sample:1 part India ink (Difco, Frankin Lakes, NJ)]. Imaging at 400X was done using a Nikon Eclipse TE200 microscope equipped with a SPOT InSight camera (SPOT Imaging, Sterling Heights, MI). Diameter measurements were made using SPOT 5.3.5 software. Additionally, KN99 and R265 were both cultured in YPD, as above, to obtain cell measurements at the time of inoculation for comparison. YPD-cultured cells did not have measurable capsule using the methods described; as such, only cell diameters were recorded.

### 2.8 Multiplex analysis

The left lobe of the lungs was snap frozen on dry ice after dissection and stored at -80°C until processing for multiplex analysis. Left lung lobes were thawed in 1 mL of PBS containing 1X protease inhibitor (Roche cOmplete, Mini Protease Inhibitor Complete) and 0.05% TritonX-100 then homogenized for 20 sec, using the OMNI homogenizer, as above. Homogenates were incubated on ice for 1 hr, with vortexing every 15 min. The samples were then centrifuged at 4°C for 20 min at 20,000xg. and the supernatants were collected, centrifuged again for 10 min, and stored at -80°C. The supernatants (50 μL samples) were measured using ProcartaPlex Mo Cytokine/Chemokine Panel I (Invitrogen) per the manufacturer’s specifications.

### 2.9 ELISA

Cell-free supernatants from mouse lungs were prepared as described above for the multiplex analysis, and IFNγ and TNFα levels were measured by ELISA according to the manufacturer’s specifications (R&D Systems, Minneapolis, MN). Fifty microliter samples were assayed.

### 2.10 Flow cytometry

Mice were vaccinated and challenged as described above. At 10 DPI, the mice were euthanized via cardiac puncture. The left lobe of the lungs was snap-frozen on dry ice and processed for ELISA, as described above. The right lung lobes were dissociated using the MACS Lung Dissociation Kit (Miltenyi) to generate a single cell suspension. Single cells were centrifuged using a 67% and 40% Percoll (Cytiva, Marlborough, MA) gradient to enrich for leukocytes, which were collected from the interphase layer. Fetal bovine serum was added to PBS at a concentration of 10% as FACS buffer. Cells were washed, resuspended in FACS buffer, and counted using the BioRad TC20. 10^6^ cells were resuspended in 100 µL of FACS buffer before the addition of CD16/CD32 (mouse FC block) at 5 μg/mL and 50 μL of BD Horizon Brilliant Stain buffer. Cells were then stained at the concentrations in **Supplementary Table S5**. After staining, cells were fixed in 2% paraformaldehyde, washed, and resuspended in FACS buffer with BioLegend Tandem Dye Stabilizer (San Diego, CA). FACS data were acquired using the 5-laser Aurora Spectral Analyzer and SpectroFlo Software (Cytek, Fremont, CA), and the spectrally unmixed data were analyzed using FlowJo V 10.8.1 (BD, Franklin Lakes, NJ). FMOs were used to draw the gates outlined in **Supplementary Figure S5**.

### 2.11 Statistics

Graphics and statistical analyses were conducted using GraphPad Prism V 10.1.1 (GraphPad Software, Boston, MA).

## 3 Results

Differences between *C. neoformans* H99 and *C. gattii* R265 infections in unvaccinated mice have been well-characterized in the literature (6, 29–33). While experimental models of cryptococcosis can vary by mouse background and inoculation method, studies from multiple research groups highlight the following consensus phenotypes for each fungal strain: i) In models of pulmonary infection, there are no significant differences in survival comparing mice infected with H99 versus R265 (6, 29, 30), ii) H99 disseminates more readily from the lungs than R265 (29, 32), iii) immune cell infiltration of the lungs is much less with R265 infection (6, 30, 31), and iv) H99 induces greater levels of Th1 and Th17 cytokines in the lungs (6, 31).

**Figure 1:**
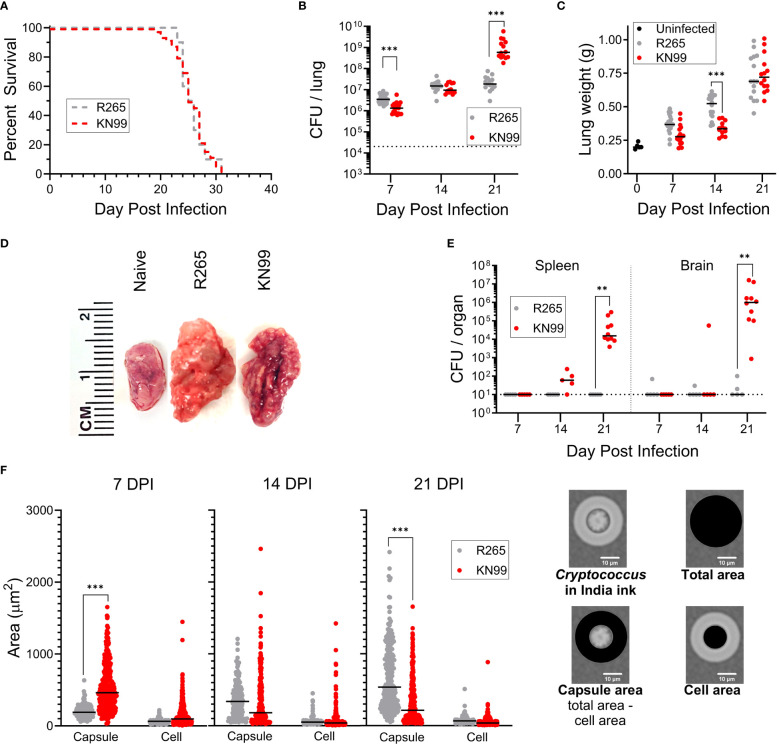
Differences between *C. neoformans* KN99 and *C. gattii* R265 in a murine model of pulmonary infection. BALB/c mice were given an orotracheal challenge of 2x10^4^ CFU of either KN99 or R265. **(A)** Survival following challenge with KN99 (*n*=50) or R265 (*n*=10). **(B)** Fungal burden in the lung over time; KN99, *n*≥13 mice/time point; and R265, *n*≥13 mice/time point. Dotted line indicates challenge dose. **(C)** Lung weight. Day 0, post infection represents uninfected mice (*n*=5); KN99, *n*≥ 13 mice/time point; and R265, *n*≥13 mice/time point. **(D)** Representative gross anatomy of the left lung lobe of infected mice at 21 DPI. **(E)** Dissemination of infection in BALB/c mice; KN99, *n*≥5 mice/time point; and R265, *n*=5 mice/time point. The dotted line indicates the lower limit of detection of 10 CFU per organ. **(F)** Size of cryptococcal cells and their capsules isolated from mouse lungs. Cell and capsule area, as depicted on the right, were calculated following measurements of the diameter of the cell plus capsule and the cell excluding capsule. For budding cells, measurements were obtained only on the mother cells. For each time point, a minimum of *n*=2 sets of lungs were imaged and measured. For all graphs, horizontal bars represent the median, and each dot represents an individual mouse **(B, C, E)** or a single cell **(F)**. Comparisons to identify significant differences were determined by: **(A)** Mantel-Cox log-rank test of Kaplan-Meier survival curves, **(B, E)** Mann Whitney test with Bonferroni correction, **(C)** unpaired, two-tailed T tests after applying the Bonferroni correction for multiple comparisons, and **(F)** one-way ANOVA with Sidak’s multiple comparisons test. Each group is the cumulative result of 2 to 6 independent experiments, with the exception of the uninfected group in **(C)** being one experiment. Not significant at *P>0.05* (non-significant comparisons are unmarked); **significant at *P ≤ 0.01*; ***significant at *P ≤ 0.001*.

In initial experiments, we sought to confirm these findings to validate our mouse model. We compared orotracheal (OT) infection of BALB/c mice with *C. neoformans* KN99 and *C. gattii* R265. There was no significant difference in survival of mice infected with either strain, with both groups succumbing to infection by 31 days post infection (DPI) (**Figure 1A**). The fungal burden in the lungs of infected mice was measured weekly during the first three weeks of infection. R265 colony forming units (CFU) were higher at 7 DPI, but by 21 DPI were surpassed significantly by KN99 (**Figure 1B**). For both strains, lung weight increased as infection progressed (**Figures 1C, D**). However, there was no difference in the weights of the lungs at 21 DPI, despite the much higher fungal burden seen in KN99-infected mice (**Figure 1C**). Substantial dissemination to the spleen and the brain occurred only for KN99-infected groups, and this was most pronounced at 21 DPI (**Figure 1E**). These trends agree with published data (6, 29, 30, 32, 33).

A shared defining feature of cryptococcal species is their distinctive capsule. This polysaccharide structure functions as a major virulence factor and has been shown to act as a shield against various aspects of the host immune response [reviewed in (34) and (35)]. We therefore isolated cryptococcal cells from infected mouse lungs and measured the diameters of the cell and cell with capsule, then calculated the cell and capsule areas for cells of each strain (**Figure 1F**). At the time of inoculation, KN99 and R265 cells cultured in YPD each had mean diameters of 5.0 μm and 5.4 μm, respectively, with resultant areas of 20 μm^2^ and 23 μm^2^ (**Supplementary Table S1**). Neither had discernible capsule by India ink staining. At 7 DPI, KN99 cells had significantly larger capsule areas than R265 cells. At 14 DPI, KN99 and R265 cells showed no difference in mean capsule area; however, total area and capsule area had significantly increased from 7 DPI for R265 and significantly decreased for KN99 (**Supplementary Tables S1**, **S2**, **Figure 1**). By 21 DPI, R265 had a significantly larger mean capsule area than KN99. At each time point, the mean cell area did not differ between KN99 and R265, therefore differences in total area of cells are attributable to changes in capsule size. Similar trends have been reported (36).

To support the changes in capsule area, carbohydrate levels in supernatants prepared from homogenized lungs were measured by the phenol sulfuric acid method. This assay has been used to estimate capsular polysaccharide generation (37). R265-infected mouse lungs had higher carbohydrate content than lungs from KN99 groups at each time point, with a significant difference between strains at 14 and 21 DPI (**Supplementary Figure S2A**). The increased capsule size for R265 correlates with the higher total carbohydrate measurements despite a lower fungal burden at 21 DPI compared to KN99. When the measured carbohydrate was normalized to the CFU in the lungs, there was more carbohydrate per R265 cell than per KN99 cell (**Supplementary Figure S2B**). This is evident in that at 21 DPI the KN99 infection in the lung had increased from the inoculum of 2x10^4^ CFU to almost 1x10^9^ CFU, while R265-infected lungs had only increased to about 2x10^7^ CFU, with no significant increase in carbohydrate production per cell between 7 DPI and 21 DPI.

Having demonstrated the KN99 and R265 phenotypes in infection models were consistent with those described in the literature, we hoped to gain insight into factors that contribute to the discordant outcomes of these infections for vaccinated mice. A better understanding of deficiencies in vaccine-mediated protection following R265 challenge could identify potential targets for vaccine modification to enhance efficacy. We therefore aimed to identify key differences in the immune response in *Cn*-*cda1*Δ*2*Δ*3*Δ-vaccinated mice by quantifying cytokines and chemokines in the lungs following fungal challenge.

It was previously shown that inoculation of *Cn-cda1*Δ*2*Δ*3*Δ directly into the lungs induced a rapid upregulation of Th1-associated cytokines in CBA/J mice (17). We expanded upon these data to determine the impact of vaccination on the response to either KN99 or R265 infection in BALB/c mice. Four different conditions at three different time points were compared: Unvaccinated, R265-challenged; unvaccinated, KN99-challenged; vaccinated, R265-challenged; and vaccinated, KN99-challenged. Each of these conditions was analyzed at 1, 3, and 7 DPI. Unvaccinated, uninfected; and vaccinated, uninfected (0 DPI) groups were included to determine baseline levels in the lungs.

**Figure 2:**
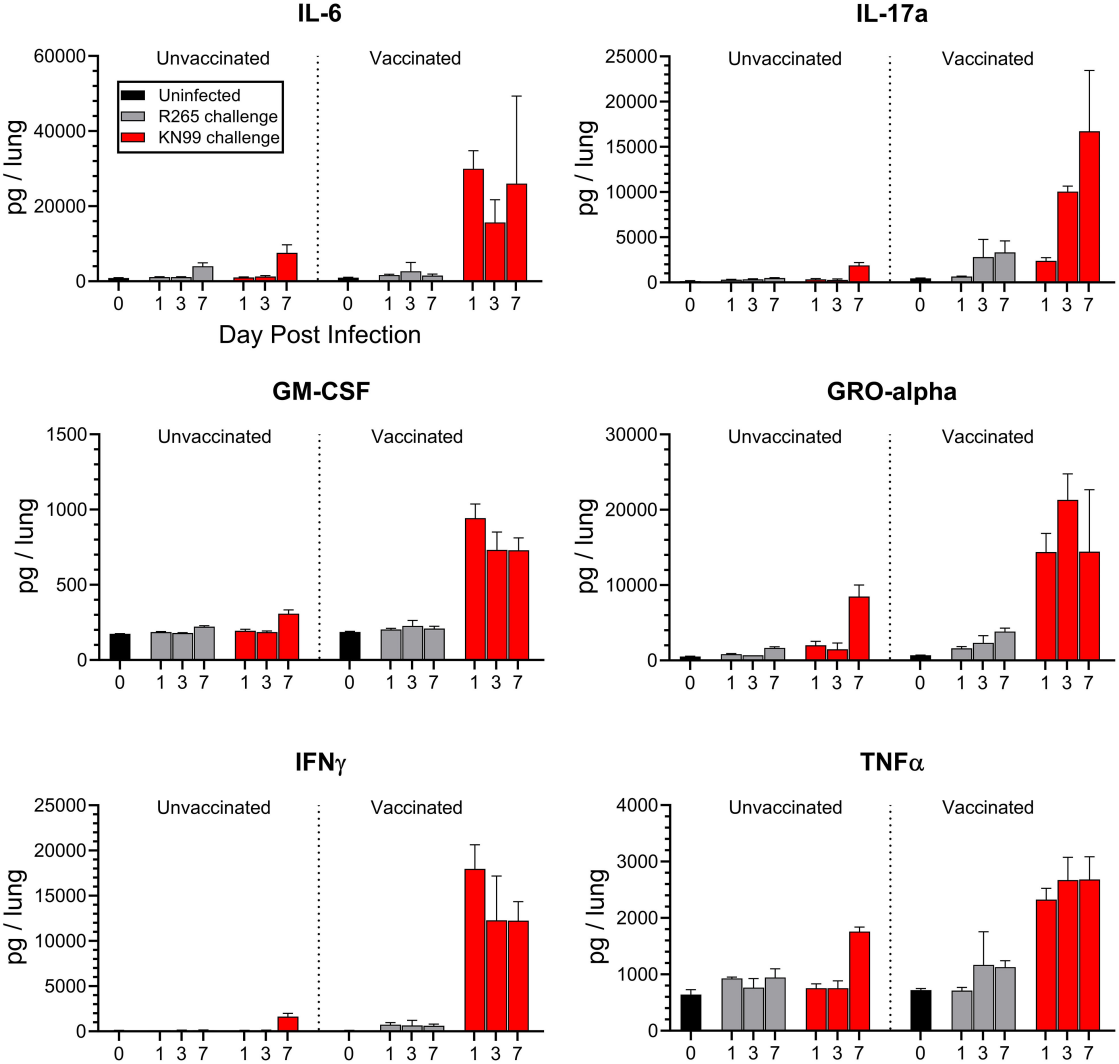
Cytokine production in the lungs of unvaccinated and vaccinated mice challenged with either KN99 or R265. BALB/c mice were vaccinated and challenged, and at the indicated time point (day post infection), the mice were euthanized, and their lungs and serum were processed for cytokine analysis via Luminex 26-plex. The Y-axis represents the total cytokine per lung, while the X-axis indicates the day on which samples were collected. Unvaccinated groups are on the left, and vaccinated groups are on the right. For each group/time point, *n*=3 mice. Bars represent the mean plus the standard error of the mean. Additional cytokines, mean cytokine levels, and statistical significance of various comparisons can be found in **Supplementary Figures S3**, **S4**; **Supplementary Tables S3**, **S4**.

Based on the literature, the importance of an early Th1-associated cytokine response in controlling infection was expected [reviewed in (38)]. Indeed, in the vaccinated, KN99-challenged mice, a group anticipated to be protected, there was an increase in production of proinflammatory cytokines as early as 1 DPI (**Figure 2**, **Supplementary Table S3**). Many of these cytokines, such as IFNγ, TNFα, GM-CSF, IL-6, IL-17a, and GRO-α, are associated with protection against fungal infections (39–44). Levels were significantly higher in vaccinated mice than in unvaccinated mice following KN99 challenge for 23 of the 26 cytokines analyzed (**Supplementary Table S4**).

Contrastingly, R265-challenged mice showed no significant differences between vaccinated and unvaccinated groups for many of the cytokines assayed. Overall, the trend was that R265 infection did not elevate cytokine levels above those seen in uninfected mice (**Figure 2**, **Supplementary Figure S3**, **Supplementary Table S4**). This was seen with both Th1- and Th2- associated cytokines. A significant increase in lung cytokine levels for vaccinated, R265-challenged mice compared to unvaccinated, R265-challenged mice was observed for only five cytokines (IL-10, Eotaxin, IP-10, MIP-1α, and RANTES). IL-10 and Eotaxin are known to be permissive of fungal growth in the lungs (45, 46). While these were elevated in vaccinated mice, it was only at one time point (7 DPI), and their levels at 7 DPI were not statistically different from the vaccinated mice given KN99 (**Supplementary Table S4**). Comparison of vaccinated mice challenged with KN99 to those challenged with R265 showed that cytokine production was higher following KN99 infection for at least one time point for most cytokines. Additionally, most cytokines in the serum were below the level of detection. However, for those that were detectable (IFNγ, IP-10, IL-6, and RANTES; **Supplementary Figure S4**), a difference from unvaccinated mice was seen only for vaccinated mice challenged with KN99, and not for those challenged with R265.

**Figure 3:**
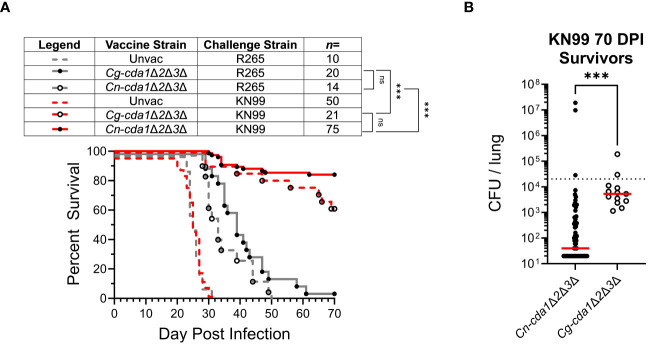
Survival following vaccination with *C. neoformans*- and *C. gattii*-derived *cda1*Δ*2*Δ*3*Δ. BALB/c mice were either unvaccinated, vaccinated with *Cg-cda1*Δ*2*Δ*3*Δ, or vaccinated with *Cn-cda1*Δ*2*Δ*3*Δ before challenge with *C. gattii* strain R265 or *C. neoformans* strain KN99. **(A)** Survival was measured until 70 DPI. All vaccinated mice had significantly prolonged survival compared with unvaccinated mice (*P*<0.001). **(B)** After 70 days, KN99-challenged survivors were euthanized, and the fungal burden in their lungs was determined (*Cn-cda1*Δ*2*Δ*3*Δ-vaccinated *n*=63, *Cg-cda1*Δ*2*Δ*3*Δ-vaccinated *n*=13). Red bars indicate the median. The lower limit of detection is 20 CFU/lung. Significance was determined by comparison of Kaplan-Meier survival curves using the Mantel-Cox, log-rank test **(A)** and the (non-parametric) Mann Whitney test **(B)**. Each group is the cumulative result of 2 to 9 independent experiments. Please note, the unvaccinated control groups reflect the same data displayed in **Figure 1A**. ns, not significant at *P>0.05*; ***significant at *P ≤ 0.001*.

Prior work showed that both *Cn-cda1*Δ*2*Δ*3*Δ and *Cg-cda1*Δ*2*Δ*3*Δ vaccines extended survival of R265-infected mice compared to unvaccinated animals (17, 22). While the ability of a *C. neoformans*-derived vaccine strain to promote survival following subsequent exposure to KN99 was demonstrated, such robust protection was not seen with R265 infection of mice vaccinated with either *C. neoformans-* or *C. gattii*-derived vaccine strains. Therefore, we questioned whether *Cg-cda1*Δ*2*Δ*3*Δ was inducing insufficient immunologic memory. We found that *Cg-cda1*Δ*2*Δ*3*Δ functions as an effective vaccine against KN99, despite their diverged phylogenetic lineages, suggesting vaccine efficacy is a product of the challenge strain, not the vaccine strain (**Figure 3A**). There was no significant difference in survival between *Cn-cda1*Δ*2*Δ*3*Δ-vaccinated and *Cg-cda1*Δ*2*Δ*3*Δ-vaccinated groups infected with KN99; these groups were equally-well protected. The same was true with R265 challenge: There was no significant difference in survival between R265-infected groups, regardless of vaccine strain. However, there was a trend favoring a survival benefit when the vaccine and challenge strains were concordant. Mean survival was 3 and 6 days longer for KN99 and R265 infections, respectively, when the vaccine strain and challenge strain shared the same genetic background. Agreeing with the benefit of a homologous vaccination-challenge system, surviving KN99-challenged mice vaccinated with *Cn-cda1*Δ*2*Δ*3*Δ had over 100X lower median CFU at the end of the study than those vaccinated with *Cg-cda1*Δ*2*Δ*3*Δ (**Figure 3B**). While the difference in CFU between groups was significant, both groups had median fungal burden in the lungs below the level of the challenge dose.

To better understand the comparatively reduced survival of *cda1*Δ*2*Δ*3*Δ-vaccinated mice challenged with R265, we considered whether the protective immune response to KN99 infection would be sufficient to induce clearance of R265. This would also allow us to examine whether R265 inhibited protection, as that would likely impact the clearance of KN99. A coinfection model was developed to try to determine whether R265 was evading or suppressing vaccine generated immunity.

**Figure 4:**
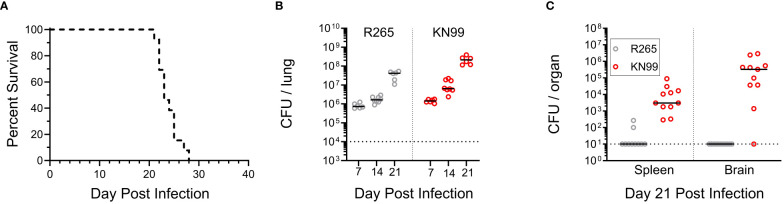
Coinfection with KN99 and R265 in unvaccinated BALB/c mice. Mice were given an orotracheal challenge of 2x10^4^ CFU, comprised of 1x10^4^ CFU of KN99 and 1x10^4^ CFU R265. Selective media was used to differentiate between strains, as described in the Methods. **(A)** Survival, *n*=13 mice. **(B)** Animals were euthanized at 7, 14, and 21 DPI to assess the fungal burden of each strain in the lungs. The horizontal dotted line represents the challenge dose of each individual strain (1x10^4^ CFU). **(C)** Dissemination to the spleen and brain at 21 DPI, horizontal dotted line represents the limit of detection of 10 CFU per organ. All data are combined from 2 to 3 independent experiments.

First, we examined whether the presence of either strain altered the phenotype of the other in unvaccinated mice. For a standard single infection, BALB/c mice received 2x10^4^ CFU; therefore, a total challenge of 2x10^4^ CFU, composed of equal parts KN99 and R265, was administered for the coinfection. To distinguish between the two strains in the coinfection, a G418-resistant strain of KN99, with wild-type virulence, was used (25). Plating on selective medium differentiated KN99 from R265 CFU during coinfection. Coinfected mice died between 21 and 28 DPI, which fell within the range of single infections with KN99 (19-31 DPI) and R265 (23-31 DPI) (**Figures 1A**, **4A**). The CFU for each strain in coinfected lungs increased over time, with KN99 being higher at each time point (**Figure 4B**). Evidence of substantial dissemination to the brain and spleen was seen for KN99, but not for R265, at 21 DPI (**Figure 4C**). Coinfection did not appear to alter the infective phenotype of either strain.

Next, the coinfection model was extended to *Cn-cda1*Δ*2*Δ*3*Δ-vaccinated mice. We established that vaccination, either with *Cn-cda1*Δ*2*Δ*3*Δ or *Cg-cda1*Δ*2*Δ*3*Δ, leads to distinct outcomes for KN99-challenged and R265-challenged mice, (**Figures 2**, **3A**) (17, 22). Vaccination allows the host to gain control of the KN99 infection in the lungs. There is an initial increase in the pulmonary fungal burden, but by 14 DPI onward there is a decreasing trend (19). Most of these mice will then survive until the study end point at 70 DPI, with a fungal burden in the lungs well below the challenge dose (19) (**Figure 3B**). However, *Cn-cda1*Δ*2*Δ*3*Δ-vaccination with R265 challenge does not typically result in any survivors at the study endpoint (17, 22). While the vaccine does extend survival of R265-challenged mice compared to those that are unvaccinated, the infection progresses to the point that all the mice die by 50 DPI.

**Figure 5:**
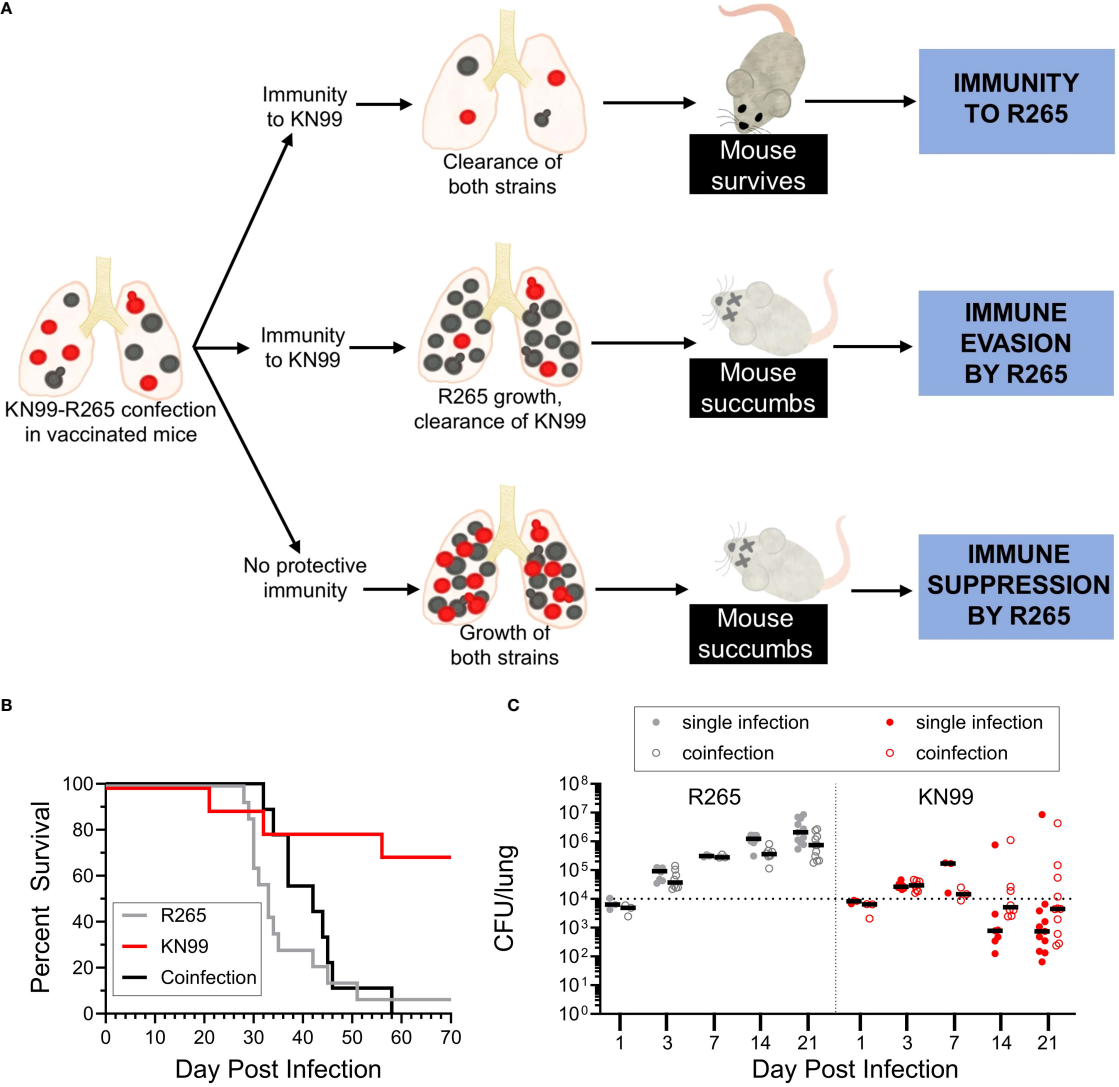
Coinfection with *C. neoformans* KN99 and *C. gattii* R265 in vaccinated mice. **(A)** Potential outcomes of coinfection in BALB/c mice vaccinated with *Cn*-*cda1*Δ*2*Δ*3*Δ. Coinfected mice were challenged as described in **Figure 4**. Mice which received a single infection of either R265 or KN99 received 1x10^4^ CFU, while coinfected mice received a total of 2x10^4^ CFU, composed of 1x10^4^ CFU of each strain. **(B)** Survival. *n*=14 mice for R265, *n*=10 mice for KN99, and *n*=9 mice for coinfection. **(C)** Animals were euthanized at the indicated time points to determine CFU. Filled circles represent mice which received a single infection, while open circles indicate the CFU of that strain isolated from coinfected lungs. Black bars represent the median. The dotted line represents the challenge dose of each individual strain (1x10^4^ CFU). For both strains, there is no statistically significant difference in singly infected lung CFU compared to the corresponding coinfected lung CFU at any of the time points analyzed, as determined by Dunn’s multiple comparisons tests following the Kruskal-Wallis Test (non-parametric one-way ANOVA). Each group is the cumulative result of 2 to 3 independent experiments, with the exception of 1 and 7 DPI in **(C)** being one experiment each.

With these strain dependent outcomes in mind, we explored whether the reduced immunity to R265 was due to immune evasion or immune suppression using KN99-R265 coinfection of *Cn-cda1*Δ*2*Δ*3*Δ-vaccinated mice. (**Figure 5A**). If a protective response to KN99 is sustained in KN99-R265 coinfection, three outcomes can be postulated. One, the proinflammatory response generated following KN99 challenge in vaccinated mice could potentially facilitate a productive vaccine response to R265. Under this scenario, the initiation of the protective immune response would be sufficient to induce clearance of R265, and act as an enhancer of the immune response to R265 (**Figure 5A**, top). With both strains cleared from the lungs, the mice would survive the coinfection. Two, in the case of immune evasion by R265 (**Figure 5A**, middle), coinfection would still allow for efficient clearance of KN99 from the lungs of vaccinated mice, and the protective, proinflammatory response would reflect that seen with a KN99 single infection. If R265 were to be completely undetected, it would continue to expand in the lungs, and the mice would still succumb to disease. Three, in the case of immune suppression (**Figure 5A**, bottom), the presence of R265 would dampen the protective response induced by KN99 in vaccinated mice. The hinderance of the proinflammatory cytokine response would inhibit protection against KN99, and one would observe KN99 continue to grow rather than see its clearance. This would also result in the death of the mice.

We first compared the mortality of vaccinated, singly infected mice to vaccinated, coinfected mice (**Figure 5B**). The survival of KN99 singly infected mice was consistent with the protection we have previously shown (**Figure 3**). The coinfected mice succumbed to infection within the same timeframe as the R265 singly infected mice. This suggests that the presence of KN99 in coinfected mice did not confer immunity to R265. The survival data alone, however, was not sufficient to differentiate between immune evasion and immune suppression by R265 (**Figure 5A**).

We therefore assessed the fungal burden in the lungs of BALB/c mice vaccinated with *Cn*-*cda1*Δ*2*Δ*3*Δ and then coinfected with KN99-R265 (**Figure 5C**). R265 in the lungs increased following challenge for both singly and coinfected groups. For each time point, there was no significant difference between R265 CFU in R265 singly infected and coinfected groups, though the coinfected mice trended slightly lower for R265 CFU than singly infected mice. With KN99 challenge, CFU in the lungs increased until 7 DPI. For subsequent time points, both singly infected and coinfected mice exhibited median KN99 CFU below the challenge dose. The KN99 CFU for the coinfected mice trended slightly higher than singly infected mice at most time points, but these differences were not statistically significant.

**Figure 6:**
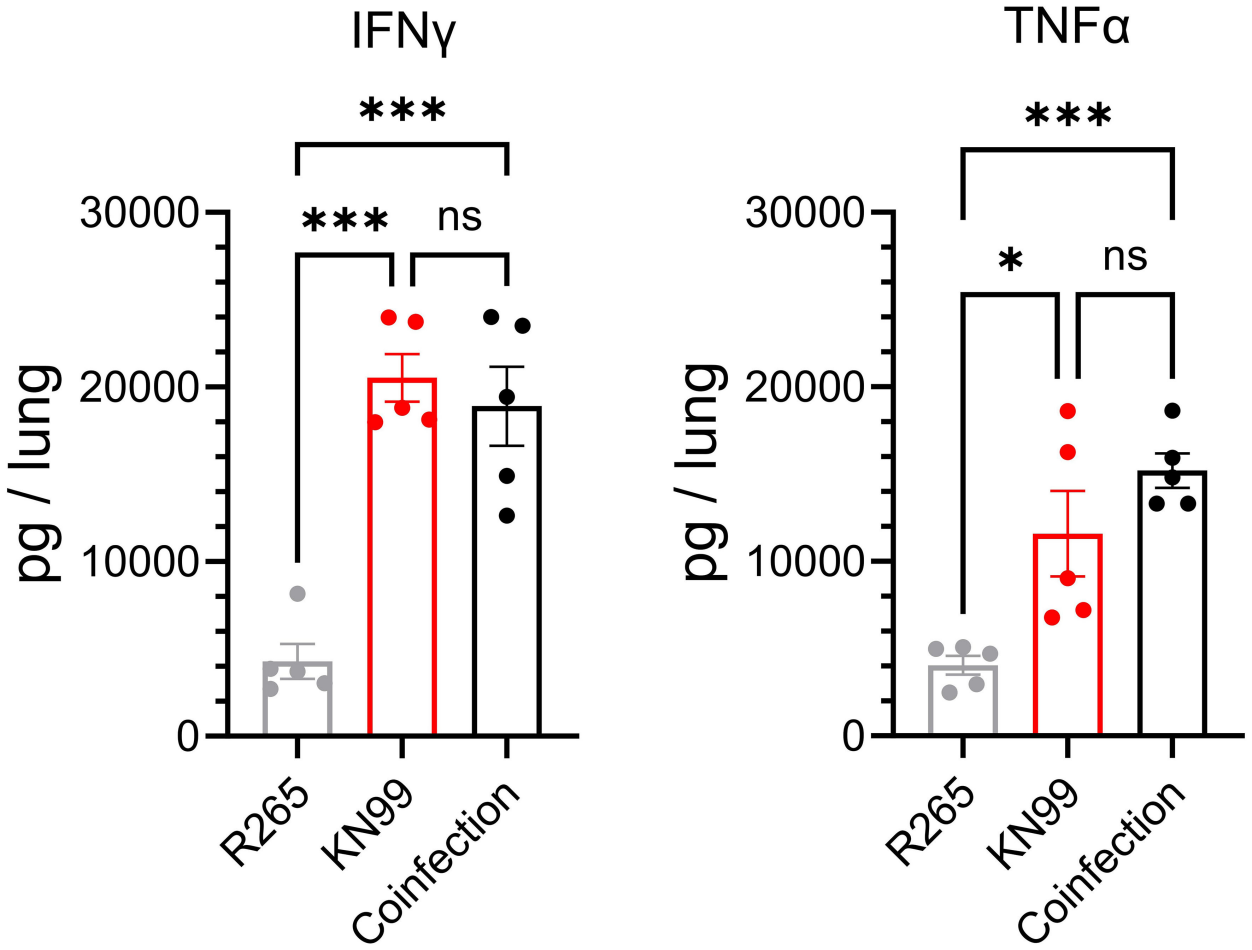
IFNγ and TNFα in the lungs of *Cn*-*cda1*Δ*2*Δ*3*Δ-vaccinated mice coinfected with KN99 and R265 at 3 DPI. Mice were vaccinated and challenged as described in **Figure 4**. Lungs were collected at 3 DPI, homogenized, and cell-free supernatants were analyzed by ELISA*; n*=5 mice per group. Each dot represents the average of two technical replicates. Significance was determined by one-way ANOVA with Sidak’s multiple comparisons test. Not significant (ns) at *P>0.05*; *significant at *P ≤ 0.05*, ***significant at *P ≤ 0.001*.

These data showed that the presence of R265 did not prevent the clearance of KN99 from the lungs of vaccinated mice, thus supporting the hypothesis that R265 evades the immune response. To further investigate this finding, cytokine responses in the lungs of vaccinated mice, which had either been singly infected or coinfected, were compared. IFNγ and TNFα, which are both critical for protection against *Cryptococcus* (41, 44), were quantified in the lungs at 3 DPI. For both IFNγ and TNFα, there were no statistical differences between the KN99-infected and the coinfected groups (**Figure 6**). Both groups were significantly elevated above the group challenged only with R265. These data suggest that during coinfection of vaccinated mice, R265 is not dampening the protective immune response that is generated to KN99.

**Figure 7:**
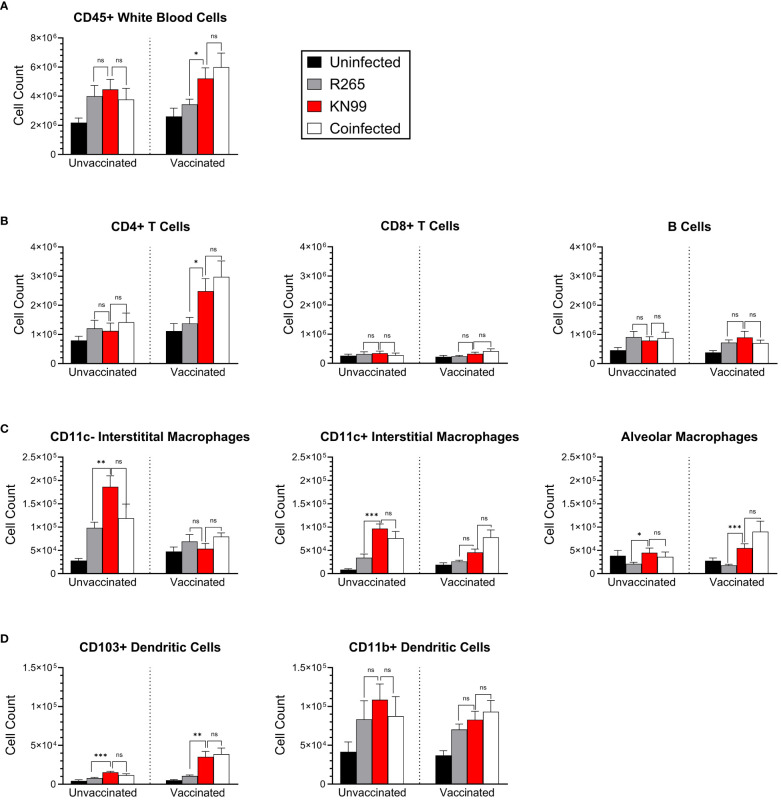
Immune cell phenotyping of lung cells in coinfected BALB/c mice. Mice were either unvaccinated (left) or vaccinated (right) with *Cn-cda1*Δ*2*Δ*3*Δ. Ten days after being infected with the indicated strain(s), the mice were euthanized, and their lungs were dissected to process for flow cytometry. Pulmonary leukocytes were isolated, and single cell suspensions were stained for expression of various surface markers (**Supplementary Table S5**). Unmixed spectral data were analyzed for the indicated cell types according to the gating strategy outlined in **Supplementary Figure S5**. **(A)** Total leukocytes. **(B)** Lymphocytes. **(C)** Macrophages. **(D)** Dendritic cells. Bars represent the mean plus the standard error of the mean. *n*=10 to 14 mice per group. Each group is the cumulative result of three experiments, with the exception of the vaccinated, unchallenged group being two experiments. Statistical significance was determined by unpaired, two-tailed t tests. Not significant (ns) at *P>0.05*; *significant at *P ≤ 0.05*, **significant at *P ≤ 0.01*, ***significant at *P ≤ 0.001*.

We also wondered whether the immunophenotype of cells in the lung reflected evasion rather than suppression following R265 infection of vaccinated mice. Flow cytometry was used to characterize immune cell recruitment to the lungs at 10 DPI, and cell populations were quantified in both unvaccinated and vaccinated mice. We adapted the staining and gating strategies for pulmonary leukocytes outlined in Yu et al., 2016 (47) (**Supplementary Table S5**, **Supplementary Figure S5**). We quantified populations and sub-populations of total leukocytes, lymphocytes, granulocytes, macrophages, dendritic cells (DCs), natural killer (NK) cells, and monocytes (**Figure 7**, **Supplementary Figure S6**).

The most distinct differences between challenge groups were seen in vaccinated mice. Total lung leukocytes were higher in KN99-infected mice than in the R265-infected mice (**Figure 7A**). The leukocyte number in the lungs of vaccinated, coinfected mice was not statistically different than that of the KN99 singly infected group. As with CFU and cytokine analysis, the vaccinated and coinfected mice displayed a phenotype very similar to the KN99 singly infected mice, despite the presence of R265 in the coinfection.

CD4+ T cells, which are critical for cryptococcal immunity, were also significantly increased in vaccinated mice which received KN99 alone or a coinfection, compared to those singly infected with R265 (**Figure 7B**). There were no differences between groups for CD8+ T cells or B cells. These data are consistent with previously established correlates of immune protection in vaccinated mice challenged with KN99, wherein protection was retained in mice deficient in either CD8+ T cells or B cells (48).

For most cell types in unvaccinated mice, KN99-infected mice were similar to R265-infected and coinfected mice. For the three macrophage subpopulations examined, however, cell counts were higher in KN99-infected and coinfected mice than in R265-infected mice (**Figure 7C**). CD11c- interstitial macrophages are associated with increased IL-10 production, which is detrimental for the clearance of cryptococcal infection (45, 49); correspondingly, CD11c- interstitial macrophages were increased in unvaccinated groups compared to vaccinated groups.

CD103+ dendritic cells have been shown to prime CD4+ T cells in a manner that skews towards a Th1 and Th17 environment (50). This type of response is thought to be beneficial in clearing cryptococcal infections from the lung (51). We saw significantly lower levels of CD103+ dendritic cells in vaccinated mice challenged with R265 than in those challenged with KN99 or coinfected (**Figure 7D**). CD11b+ DCs have been shown to promote either Th17 or Th2 differentiation, depending on their maturation state, but no significant differences were seen between groups for this DC subset (52). These FACS data demonstrate similarities in recruitment of protection-associated immune cells between vaccinated mice given KN99 alone or a coinfection, and a clear difference between these groups and vaccinated mice challenged with R265. This further supports an immune evasion phenotype of R265 in *Cn-cda1*Δ*2*Δ*3*Δ-vaccinated mice.

## 4 Discussion

In combination with previous work (17, 22), we demonstrated that the live *cda1*Δ*2*Δ*3*Δ vaccines protect KN99-infected mice significantly better than R265-infected mice. Here we present cryptococcal coinfection of vaccinated mice, wherein simultaneous challenge with both KN99 and R265 serves as a novel tool to examine strain-dependent host-pathogen interactions. Modeling KN99-R265 coinfection allows for discrimination between immune evasion and immune suppression. As with single infections of vaccinated mice, protective immunity against KN99 and comparatively attenuated protection against R265 was observed during coinfection. The presence of R265 did not dampen the ability of vaccinated mice to clear KN99 from the lungs. This indicates immune evasion by *C. gattii* R265 because the protection-associated immune response to KN99 was unaffected by coinfection.

Many human pathogens have evolved to either evade or suppress host immunity. Cytomegalovirus (CMV), for example, evades innate immunity through the production of miRNAs that prevent recognition and subsequent killing of infected host cells by NK cells (53). This evasion, however, can have downstream suppressive affects through the alteration of NK signaling. Direct suppression of the innate immune response by CMV can also be mediated by viral miRNAs. Certain CMV miRNAs are able to bind host cytokines, which in turn reduces the local cytokine concentration and halts chemotactic recruitment of immune cells, leading to an overall dampened response (53).

Evasion and suppression of immunity by pathogens may also occur during the adaptive immune response. Consider Hepatitis B, a virus known to mutate its surface antigen genes, which changes the viral envelope and facilitates escape of vaccine-mediated immunity (54). In this case, modified surface antigens not binding efficiently to vaccine-induced antibodies allows Hepatitis B to evade this arm of the adaptive immune response (54). Suppression of adaptive immunity can also be seen following vaccination. For example, *Streptococcus pneumoniae* infection following influenza is common in humans. In a mouse model, vaccination against *S. pneumoniae* allows for 10,000-fold better clearance of bacterial burden in the lungs compared to unvaccinated mice (55). However, a sublethal dose of influenza prior to *S. pneumoniae* challenge renders bacterial levels in the lungs of vaccinated mice comparable to levels seen in unvaccinated groups (55). The ablation of clearance of *S. pneumoniae*, despite vaccine efficacy in a single infection, demonstrated suppression of the adaptive immune response by the Influenza virus.

In the context of a vaccination model of R265 infection, clear delineations between immune suppression and immune evasion, such as those described above, had not yet been established. However, immune responses to various R265-coinfections in unvaccinated mice have been described. For example, both Influenza A virus and the nematode *Strongyloides venezuelensis* exacerbated the pathology of R265 infections. Administration of either virus or nematode prior to infection with *C. gattii* R265 worsened outcomes in mice significantly, based on decreased survival and reduced phagocytic killing of R265, resulting in increased fungal growth (56, 57). Contrastingly, primary exposure to either *Pseudomonas aeruginosa* or *Staphylococcus aureus* enhanced immunity to subsequent R265 infection, such that mean survival was extended, and fungal growth was better controlled than in an R265 single infection (58, 59). R265 can also modulate the severity of coinfection; when *C. gattii* was administered prior to *S. pneumoniae* challenge, coinfected mice not only had a higher pulmonary bacterial burden than singly infected mice, but they also showed significantly more dissemination to the spleen, liver, and brain (60).

Coinfections comprised specifically of cryptococcal species have also been characterized experimentally. Such studies have shown that one strain of *Cryptococcus* can greatly impact the outcome for the coinfected strain of *Cryptococcus*. For example, coinfection with strains of KN99 bearing a and α mating types highlighted alterations in pathogenicity when both strains were introduced to the lungs. During single infections, KN99a and KN99α formed titan cells and disseminated to the brain equally (61). However, during coinfection of the two mating types, titan cell formation by KN99a was increased, which inhibited its dissemination (61). Modulation of pathogenicity of coinfected strains by *C. gattii* has also been documented. *Ex vivo* R265-coinfection of macrophages with *C. gattii* non-outbreak strains led to increased intracellular proliferation rates of the non-outbreak strains (62). Such an impact by R265 has also been seen *in vivo*. In unvaccinated C57BL/6 mice, intranasal coinfection with H99 and R265 yielded decreased transcription of Th1-attracting chemokines compared to H99 alone, and the magnitude of this difference depended on the R265 dosage (31). While the clinical relevance of cryptococcal coinfections is unclear, a mouse model of coinfection is a unique immunological tool, particularly following vaccination. It provides a perspective to answer the questions of whether the mode(s) of immune avoidance is consistent between both the innate and adaptive immune responses, and whether immune evasion and immune suppression can be clearly differentiated.

In the KN99-R265 coinfection presented here, vaccinated and coinfected mice mirrored the immunophenotype of KN99 singly infected mice. Therefore, R265 was not suppressing the protective immune response to KN99 in vaccinated, coinfected mice. Fungal clearance, cytokine production, and immune cell populations in the lungs were the metrics by which immune suppression and evasion were gauged. The resultant immune evasion phenotype by R265 was reinforced through each of these criteria. In vaccinated, coinfected mice, the fungal burden in the lungs represented by either strain was consistent with what we saw with single infections. There was no difference in the lung CFU from singly infected mice compared to the corresponding strain from coinfected mice at any time point for either KN99 or R265. KN99 was as efficiently cleared from the lungs of mice during coinfection as in KN99 single infection. Although fungal killing of KN99 was not inhibited by the presence of R265, KN99 was unable to promote clearance of R265. Production of IFNγ and TNFα in the coinfected lungs was significantly higher than in R265 singly infected lungs, and no different from levels measured for KN99 singly infected lungs. Yet, this ongoing proinflammatory immune response did not have any impact on R265. This pattern of immunophenotypes shared by KN99 singly infected and coinfected mice starkly contrasting R265 singly infected mice was also seen with CD45+ leukocyte, CD4+ T cell, alveolar macrophage, and CD103+ dendritic cell numbers in the lungs. These trends are supportive of immune evasion by R265.

The evidence of immune evasion demonstrated by these experiments is consistent with the recently documented phenomenon of dendritic cell immunoparalysis by R265 (63). Unlike with H99, DC uptake of *C. gattii* does not induce DC activation or maturation, and consequently phagocytosis does not lead to antigen presentation (64, 65). This defect has been attributed to the lack of TNFα signaling by DCs in the presence of *C. gattii* (65) and poses a challenge for vaccine development. However, it has been shown that T cell responses can be restored *in vitro* by treating DCs with maturation cytokines (65). In addition, DC immunoparalysis can be negated by disrupting F-actin cages within DCs by treatment with cytochalasin-D (63). Combination therapy, such as treatment with recombinant cytokines, may be one approach to consider in the future.

While both KN99 and R265 are commonly used in cryptococcal research to characterize vaccine responses it is important to note that these strains are hypervirulent which lends well to clear outcomes for evaluating vaccine efficacy. Although R265 is an outbreak strain in endemic areas such as the Pacific Northwest of the United States and in British Columbia, Canada, it may not be representative of other *C. gattii* strains found elsewhere in the world. Many non-outbreak clinical isolates of *C. gattii* are less virulent than R265 (27, 66). We have shown that Australian environmental isolate of *C. gattii*, strain WM276, was of comparable virulence to R265 in unvaccinated CBAJ mice, and that vaccination with heat-killed *Cn-cda1*Δ*2*Δ*3*Δ rendered similar delayed mortality over unvaccinated controls as R265 (17). Future studies will inform whether *C. gattii*-derived vaccine strains can improve survival, and whether more robust protection is possible against less virulent strains.

Variations in antigenicity between *Cn*- and *Cg*-derived strains have not yet been studied. However, each Cda has been characterized extensively as recombinant protein vaccines (19, 48, 67–69). At the protein level, H99 and R265 Cda1, Cda2, and Cda3 are 85%, 83%, and 85% identical, respectively (22). Despite substantial sequence similarity in these enzymes, the protective immunity generated by *Cn*-derived recombinant Cda vaccination was less with R265 challenge than with KN99 (19). The impact of protein expression *in vivo* may induce important strain-dependent differences. R265 makes twice the amount of cell wall chitosan in a mouse model of infection (22). Additionally, the predominant Cda contributing to chitosan production differs between KN99 (Cda1) and R265 (Cda3) (22). While capsular differences undoubtedly play a role in the disparate vaccine outcomes between strains, it could also be that chitosan production is equally important. It is worth considering that both *Cn-cda1*Δ*2*Δ*3*Δ and *Cg-cda1*Δ*2*Δ*3*Δ, are capsule-producing strains *in vivo*. Also of interest, heat-killed KN99 that had been grown in yeast nitrogen base medium buffered to pH 7 also provided protection against a subsequent challenge (70). Despite the well-established immunosuppressive nature of the cryptococcal capsule, both the live-attenuated strains and the heat-killed WT strain, which are inoculated directly into the lungs of mice, generated immunity to KN99 challenge.

The allocation of cellular resources to either generating larger amounts of capsule per cell by R265 or to increased proliferation by KN99 may contribute to the different pathologies and vaccine responses exhibited by these strains. It is important to note that R265 exhibits no deficit in colonization of extrapulmonary organs when administered intravenously rather than delivered into the lungs (29). The factors driving a dissemination phenotype for KN99, but not for R265 remain speculative. One possibility is a threshold for fungal burden in the lungs must be reached before dissemination can occur, and infection with R265 does not reach that level before the mice succumb. However, arguing against this possibility is fungal burdens for the two strains are similar at 14 DPI when dissemination of KN99 is already underway. Vasculature in the lungs may also play a role in limiting dissemination of R265. Increased total cell area is a product of capsule thickness for this strain: The size of the R265 cell does not significantly change over the course of infection but capsule size increases steadily over time. If the capsule renders these cells too large to exit the lungs through the bloodstream, they cannot have the direct interactions with brain endothelial cells that would otherwise allow for entrance into the brain (29). Moreover, hypercapsular strains of *C. neoformans* have been shown to be less neurovirulent than hypocapsular strains (71). Yeast cells with larger capsule sizes are also less susceptible to phagocytosis (72), which could reduce crossing of the blood-brain barrier via the Trojan horse mechanism [reviewed in (73)].

In summary, we developed a novel vaccination model of *C. neoformans-C. gattii* coinfection in mice. With this model, we demonstrated that *C. gattii* was evading, rather than suppressing, the adaptive immune response to infection. Future *C. gattii* vaccine design should include strategies to elicit protective responses that overcome immune evasion. Use of a well-characterized strain like KN99 as an internal control for co-infections was key to our ability to differentiate between immune suppression and evasion. Ideally, one would also want to view the host response at physically separated focal points of KN99 and R265 infection within lung tissue. The utilization of spatial transcriptomic technologies could augment our current perspective by documenting what are the localized host responses in the lung that distinguish R265 and KN99 colonization.

## Data availability statement

The raw data supporting the conclusions of this article will be made available by the authors, without undue reservation.

## Ethics statement

The animal study was approved by University of Massachusetts Chan Medical School Institutional Use and Care of Animals Committee. The study was conducted in accordance with the local legislation and institutional requirements.

## Author contributions

MH: Writing – original draft, Writing – review & editing, Conceptualization, Investigation, Formal analysis, Methodology. DC: Investigation, Writing – review & editing. JL: Funding acquisition, Resources, Writing – review & editing. SL: Conceptualization, Formal analysis, Funding acquisition, Project Administration, Resources, Supervision, Writing – review & editing. CS: Conceptualization, Methodology, Investigation, Formal analysis, Funding acquisition, Project administration, Resources, Supervision, Writing – review & editing.
