# Supplementary material for: Immune evasion by Cryptococcus gattii in vaccinated mice coinfected with C. neoformans
Source: Front Immunol. 2024 Feb 26;15:1356651. doi: 10.3389/fimmu.2024.1356651 (PMC10925662; doi:10.3389/fimmu.2024.1356651)
Supplement: Supplementary file 1 [file DataSheet_1.pdf]

Supplementary Material

Table S1: Values derived from statistical analysis of cell and capsule measurements.

	R265									
	7 DPI n=127			14 DPI n=170			21 DPI n=250			YPD n=62
Areas (μm^2)	Total	Cell	Capsule	Total	Cell	Capsule	Total	Cell	Capsule	Cell
Minimum	79	20	59	73	19	47	73	23	50	11
Maximum	853	218	635	1,446	452	1,208	2,925	509	2,416	35
Median	254	64	188	396	51	339	603	66	538	23
Mean	272	66	206	437	67	370	748	77	671	23
Std. Deviation	115	33	91	271	58	247	488	46	456	5
Std. Error of Mean	10	3	8	21	4	19	31	3	29	1
	KN99									
	7 DPI n=349			14 DPI n=191			21 DPI n=270			YPD n=41
Areas (μm^2)	Total	Cell	Capsule	Total	Cell	Capsule	Total	Cell	Capsule	Cell
Minimum	41	7	32	29	5	23	38	7	31	9
Maximum	2,968	1,448	1,652	2,781	1,425	2,461	2,088	885	1,656	35
Median	572	97	462	230	38	180	247	37	213	20
Mean	690	154	536	445	99	346	396	59	337	20
Std. Deviation	447	162	326	514	182	374	370	69	317	5
Std. Error of Mean	24	9	17	37	13	27	23	4	19	1

Table S2: Statistical significance of changes in cell, capsule, and total area measurements over time for cryptococcal cells from lungs of infected mice.

Strain	Group 1 Area	Group 2 Area	P value	Significance
R265	7 DPI total	14 DPI total	0.0002	***
	14 DPI total	21 DPI total	<0.0001	***
	7 DPI cell	14 DPI cell	>0.9999	ns
	14 DPI cell	21 DPI cell	>0.9999	ns
	7 DPI capsule	14 DPI capsule	0.0002	***
	14 DPI capsule	21 DPI capsule	<0.0001	***
KN99	7 DPI total	14 DPI total	<0.0001	***
	14 DPI total	21 DPI total	0.8935	ns
	7 DPI cell area	14 DPI cell	0.6922	ns
	14 DPI cell	21 DPI cell	0.9820	ns
	7 DPI capsule	14 DPI capsule	<0.0001	***
	14 DPI capsule	21 DPI capsule	>0.9999	ns

*Significance was determined by one-way ANOVA with Sidak's multiple comparisons test. ns, not significant at P>0.05; *** significant at P≤0.001.*

Table S3: Mean ± SEM of cytokines (pg/lung).

		Unvaccinated							Vaccinated						
		none	R265			KN99			none	R265			KN99		
	Cytokine	0 DPI	1 DPI	3 DPI	7 DPI	1 DPI	3 DPI	7 DPI	0 DPI	1 DPI	3 DPI	7 DPI	1 DPI	3 DPI	7 DPI
Lungs	IL-1β	100 ± 7	122 ± 3	115 ± 13	135 ± 15	121 ± 9	115 ± 18	486 ± 41	108 ± 4	156 ± 17	399 ± 174	440 ± 53	888 ± 41	2591 ± 510	2484 ± 751
	IL-2	132 ± 14	173 ± 4	162 ± 16	189 ± 15	136 ± 3	247 ± 55	266 ± 16	137 ± 8	158 ± 1	410 ± 185	196 ± 44	940 ± 75	336 ± 57	255 ± 8
	IL-4	63 ± 3	74 ± 2	68 ± 3	155 ± 9	82 ± 6	68 ± 4	2364 ± 745	80 ± 6	142 ± 20	476 ± 199	776 ± 170	7154 ± 2558	4901 ± 12	1887 ± 203
	IL-5	98 ± 156	149 ± 1	131 ± 17	340 ± 51	172 ± 26	151 ± 35	1247 ± 647	119 ± 8	155 ± 15	236 ± 63	436 ± 150	951 ± 142	1281 ± 295	699 ± 31
	IL-6	882 ± 107	1133 ± 71	1143 ± 101	4015 ± 919	1024 ± 171	1289 ± 259	7574 ± 2134	978 ± 96	1676 ± 174	2696 ± 1351	1538 ± 381	29973 ± 4800	15696 ± 6028	26037 ± 23298
	IL-9	2716 ± 247	3690 ± 13	3458 ± 500	4133 ± 538	3305 ± 283	3271 ± 705	6302 ± 161	3056 ± 119	2704 ± 39	5042 ± 1625	4163 ± 1281	4010 ± 335	7692 ± 1150	5862 ± 231
	IL-10	595 ± 64	994 ± 32	815 ± 111	790 ± 12	838 ± 38	830 ± 150	1031 ± 1034	723 ± 82	671 ± 72	882 ± 82	1281 ± 37	720 ± 123	1409 ± 209	1351 ± 113.5
	IL-12p70	169 ± 29	309 ± 12	224 ± 20	323 ± 51	254 ± 21	224 ± 42	699 ± 167	261 ± 93	214 ± 76	397 ± 216	193 ± 72	746 ± 58	620 ± 173	924 ± 178
	IL-13	161 ± 30	265 ± 12	234 ± 45	332 ± 54	259 ± 22	223 ± 69	1513 ± 660	202 ± 13	209 ± 15	381 ± 119	550 ± 219	835 ± 121	1316 ± 233	779 ± 97
	IL-17a	166 ± 41	310 ± 30	342 ± 72	487 ± 40	340 ± 82	285 ± 98	1895 ± 308	447 ± 43	656 ± 40	2805 ± 1124	3332 ± 1270	2386 ± 347	10057 ± 596	16717 ± 6731
	IL-18	6034 ± 86	6506 ± 44	6558 ± 75	8294 ± 256	11700 ± 4721	6216 ± 442	21420 ± 2552	6299 ± 60	6951 ± 239	9632 ± 1727	10879 ± 1314	43901 ± 9557	36617 ± 6681	29929 ± 1949
	IL-22	1212 ± 15	1306 ± 21	1259 ± 27	1288 ± 8	1272 ± 59	1265 ± 8	1524 ± 36	1268 ± 33	1326 ± 44	1921 ± 489	1475 ± 162	2562 ± 349	3755 ± 587	2445 ± 549
	IL-23	402 ± 7	445 ± 11	487 ± 60	495 ± 25	461 ± 49	462 ± 54	600 ± 43	396 ± 6	416 ± 22	452 ± 50	443 ± 46	417 ± 10	529 ± 41	485 ± 6
	IL-27	434 ± 32	415 ± 14	465 ± 52	483 ± 43	416 ± 41	933 ± 169	563 ± 21	412 ± 16	381 ± 25	609 ± 133	432 ± 101	462 ± 7	677 ± 121	562 ± 4
	Eotaxin	24735 ± 2280	26763 ± 819	30589 ± 2037	27692 ± 3278	45642 ± 12530	25073 ± 3974	58585 ± 5395	23601 ± 1341	33091 ± 1611	40324 ± 2158	60503 ± 1547	54145 ± 6206	66926 ± 4486	45570 ± 223
	GM-CSF	175 ± 1	186 ± 4	180 ± 2	221 ± 7	195 ± 10	186 ± 7	308 ± 25	187 ± 4	204 ± 7	227 ± 21	209 ± 15	944 ± 93	732 ± 119	730 ± 82.4
	GRO-alpha	521 ± 45	832 ± 68	680 ± 5	1657 ± 156	2004 ± 524	1490 ± 829	8494 ± 1522	698 ± 24	1610 ± 227	2328 ± 561	3827 ± 464	14376 ± 2495	21322 ± 3435	14440 ± 8221
	IFNγ	100 ± 16	140 ± 2	107 ± 22	142 ± 21	104 ± 14	108 ± 20	1628 ± 371	107 ± 11	739 ± 220	665 ± 320	610 ± 191	17974 ± 2671	12284 ± 4900	12235 ± 2113
	IP-10	2068 ± 159	2796 ± 157	3023 ± 425	5672 ± 103	6542 ± 2278	4589 ± 1579	52371 ± 5730	3362 ± 480	34024 ± 2376	26877 ± 8093	41649 ± 9177	67907 ± 1850	73297 ± 2663	58989 ± 1317
	MCP-1	2094 ± 68	2215 ± 35	2462 ± 201	7559 ± 921	4237 ± 1025	7792 ± 4658	25781 ± 3114	2706 ± 466	5785 ± 507	7151 ± 1977	11385 ± 2380	30825 ± 433	35256 ± 4606	27976 ± 3876
	MCP-3	532 ± 53	633 ± 20	674 ± 77	3545 ± 903	1912 ± 610	1525 ± 692	23743 ± 2004	651 ± 72	4794 ± 948	4653 ± 1566	8953 ± 1894	26425 ± 900	29136 ± 3640	19868 ± 1072
	MIP-1α	5295 ± 420	9156 ± 492	9990 ± 635	26270 ± 3622	19472 ± 4437	11227 ± 3056	173971 ± 5963	44521 ± 12569	49741 ± 5171	126101 ± 18248	134606 ± 11651	344442 ± 30385	361369 ± 52821	382905 ± 50239
	MIP-1β	125 ± 18	221 ± 14	278 ± 18	765 ± 76	439 ± 119	308 ± 60	7215 ± 467	479 ± 50	1332 ± 162	3215 ± 967	3182 ± 646	11307 ± 1572	18020 ± 4707	14387 ± 2156
	MIP-2	100 ± 30	260 ± 57	180 ± 34	229 ± 21	315 ± 97	197 ± 75	1306 ± 318	285 ± 27	848 ± 384	1366 ± 467	690 ± 67	3829 ± 786	3807 ± 761	4833 ± 1343
	Rantes	8627 ± 827	11272 ± 999	14751 ± 1141	18468 ± 633	18620 ± 4351	11095 ± 957	53629 ± 5647	11752 ± 919	22292 ± 1548	31977 ± 8413	61738 ± 10437	48424 ± 2923	98720 ± 8546	87897 ± 45
	TNFα	641 ± 89	930 ± 22	765 ± 160	943 ± 154	756 ± 76	756 ± 130	1759 ± 78	721 ± 27	710 ± 59	1168 ± 340	1130 ± 110	2324 ± 202	2672 ± 403	2682 ± 403
Serum	IL-6	47 ± 1	46 ± 1	47 ± 1	51 ± 1	48 ± 1	85 ± 20	55 ± 3	47 ± 1	51 ± 2	50 ± 1	52 ± 3	61 ± 1	85 ± 18	507 ± 120
	IFNγ	61 ± 4	55 ± 4	51 ± 7	89 ± 8	49 ± 4	78 ± 8	139 ± 45	61 ± 14	71 ± 9	120 ± 25	81 ± 13	1239 ± 314	1644 ± 523	2277 ± 560
	IP-10	152 ± 19	163 ± 13	162 ± 5	249 ± 35	157 ± 11	244 ± 98	775 ± 84	195 ± 12	187 ± 21	199 ± 56	238 ± 41	1580 ± 242	1325 ± 63	2963 ± 93
	Rantes	18 ± 7	32 ± 9	26 ± 6	32 ± 6	33 ± 5	9 ± 9	57 ± 11	25 ± 5	13 ± 7	37 ± 9	41 ± 15	66 ± 28	108 ± 44	189 ± 2

Table S4: Statistical significance of cytokine and chemokines graphed in Figure 2 and Figure S3, determined by one-way ANOVA and Sidak's multiple comparisons test.

	Groups	R265 unvac vs R265 vac			KN99 unvac vs KN99 vac			R265 vac vs KN99 vac		
	DPI	1	3	7	1	3	7	1	3	7
Lung cytokines	IL-1B	>0.9999<br>ns	0.9843<br>ns	0.9502<br>ns	0.0939<br>ns	<0.0001<br>***	<0.0001<br>***	0.1237<br>ns	<0.0001<br>***	<0.0001<br>***
	IL-2	>0.9999<br>ns	0.2299<br>ns	>0.9999<br>ns	<0.0001<br>***	0.9915<br>ns	>0.9999<br>***	<0.0001<br>***	0.9947<br>ns	0.9996<br>ns
	IL-4	>0.9999<br>ns	>0.9999<br>ns	0.9999<br>ns	0.0002<br>***	0.0247<br>*	>0.9999<br>ns	0.0002<br>***	0.0205<br>*	0.995<br>ns
	IL-5	>0.9999<br>ns	>0.9999<br>ns	>0.9999<br>ns	0.2111<br>ns	0.0471<br>*	0.76<br>ns	0.1914<br>ns	0.037<br>*	0.9972<br>ns
	IL-6	>0.9999<br>ns	>0.9999<br>ns	>0.9999<br>ns	0.0035<br>**	0.5025<br>ns	0.2077<br>ns	0.0044<br>**	0.4911<br>ns	0.039<br>*
	IL-9	0.9867<br>ns	0.8837<br>ns	>0.9999<br>ns	0.9989<br>ns	0.0155<br>*	>0.9999<br>ns	0.9243<br>ns	0.2076<br>ns	0.8371<br>ns
	IL-10	0.2657<br>ns	>0.9999<br>ns	0.0498<br>*	0.9922<br>ns	0.0141<br>*	0.4135<br>ns	>0.9999<br>ns	0.0122<br>*	>0.9999<br>ns
	IL-12p70	0.9994<br>ns	0.9771<br>ns	0.9926<br>ns	0.0488<br>*	0.2904<br>ns	0.8934<br>ns	0.0277<br>*	0.8243<br>ns	0.0045<br>**
	IL-13	>0.9999<br>ns	>0.9999<br>ns	0.9986<br>ns	0.5982<br>ns	0.0661<br>ns	0.4291<br>ns	0.4921<br>ns	0.0879<br>ns	0.9991<br>ns
	IL-17a	>0.9999<br>ns	0.9289<br>ns	0.7564<br>ns	0.9535<br>ns	0.0012<br>**	<0.0001<br>***	0.9839<br>ns	0.0082<br>**	<0.0001<br>***
	IL-18	>0.9999<br>ns	0.9999<br>ns	>0.9999<br>ns	0.0001<br>***	0.0009<br>***	0.8605<br>ns	<0.0001<br>***	0.001<br>**	0.0616<br>ns
	IL-22	>0.9999<br>ns	0.7753<br>ns	>0.9999<br>ns	0.0357<br>*	0.0001<br>***	0.381<br>ns	0.0484<br>*	0.0014<br>**	0.3171<br>ns
	IL-23	0.9997<br>ns	0.9993<br>ns	0.9739<br>ns	0.9902<br>ns	0.929<br>ns	0.4231<br>ns	>0.9999<br>ns	0.7734<br>ns	0.9975<br>ns
	IL-27	>0.9999<br>ns	0.9065<br>ns	0.9999<br>ns	>0.9999<br>ns	0.3118<br>ns	>0.9999<br>ns	0.9952<br>ns	0.9988<br>ns	0.9477<br>ns
	Eotaxin	0.9899<br>ns	0.9255<br>ns	0.0065<br>**	0.9347<br>ns	0.0005<br>***	0.7127<br>ns	0.0837<br>ns	0.0155<br>*	0.6609<br>ns
	GM-CSF	>0.9999<br>ns	0.9987<br>ns	>0.9999<br>ns	<0.0001<br>***	<0.0001<br>***	<0.0001<br>***	<0.0001<br>***	<0.0001<br>***	<0.0001<br>***
	GRO-α	>0.9999<br>ns	0.9998<br>ns	0.9962<br>ns	0.003<br>**	<0.0001<br>***	0.5295<br>ns	0.0022<br>**	<0.0001<br>***	0.0317<br>*
	IFNγ	>0.9999<br>ns	>0.9999<br>ns	>0.9999<br>ns	<0.0001<br>***	0.0007<br>***	0.0032<br>**	<0.0001<br>***	0.0003<br>***	0.0011<br>**
	IP-10	0.0005<br>***	0.0234<br>*	<0.0001<br>***	<0.0001<br>***	<0.0001<br>***	0.9806<br>ns	0.0002<br>***	<0.0001<br>***	0.1793<br>ns
	MCP-1	0.9498<br>ns	0.8794<br>ns	0.9255<br>ns	<0.0001<br>***	<0.0001<br>***	0.9993<br>ns	<0.0001<br>***	<0.0001<br>***	0.0015<br>**
	MCP-3	0.5252<br>ns	0.7139<br>ns	0.2109<br>ns	<0.0001<br>***	<0.0001<br>***	0.7417<br>ns	<0.0001<br>***	<0.0001<br>***	0.0027<br>**
	MIP-1α	0.8772<br>ns	0.0286<br>*	0.0205<br>*	<0.0001<br>***	<0.0001<br>***	<0.0001<br>***	<0.0001<br>***	<0.0001<br>***	<0.0001<br>***
	MIP-1B	0.9999<br>ns	0.9385<br>ns	0.9624<br>ns	0.001<br>**	<0.0001<br>***	0.095<br>ns	0.0026<br>**	<0.0001<br>***	0.0025<br>**
	MIP-2	0.9869<br>ns	0.6921<br>ns	0.9978<br>ns	0.0002<br>***	0.0007<br>***	0.0009<br>***	0.0017<br>**	0.0119<br>*	0.0001<br>***
	RANTES	0.8142<br>ns	0.4296<br>ns	0.0001<br>***	0.0086<br>**	<0.0001<br>***	0.0066<br>**	0.0261<br>*	<0.0001<br>***	0.0581<br>ns
	TNFα	>0.9999<br>ns	0.9843<br>ns	0.9502<br>ns	0.0002<br>***	<0.0001<br>***	0.082<br>ns	0.0001<br>***	0.0003<br>***	0.0009<br>***
Serum cytokines	IL-6	>0.9999<br>ns	>0.9999<br>ns	>0.9999<br>ns	>0.9999<br>ns	>0.9999<br>ns	<0.0001<br>***	>0.9999<br>ns	0.9509	<0.0001<br>***
	IFNγ	>0.9999<br>ns	>0.9999<br>ns	>0.9999<br>ns	0.0068<br>**	0.0013<br>**	<0.0001<br>***	0.008<br>**	0.0005<br>***	<0.0001<br>***
	IP-10	>0.9999<br>ns	>0.9999<br>ns	>0.9999<br>ns	<0.0001<br>***	<0.0001<br>***	<0.0001<br>***	<0.0001<br>***	<0.0001<br>***	<0.0001<br>***
	RANTES	0.9967<br>ns	>0.9999<br>ns	>0.9999<br>ns	0.8766<br>ns	0.0189<br>*	0.0012<br>**	0.3563<br>ns	0.0928<br>ns	0.0003<br>***

*ns not significant at $P>0.05$; * significant at $P\leq0.05$; ** significant at $P\leq0.01$; *** significant at $P\leq0.001$.*

Table S5: FACS Panel.

Marker	Fluorophore	Manufacturer	Cat#	Staining concentration (µg/mL)
**CD45**	AF488	BioLegend	103121	2.5
**CD4**	APC	BD	553051	2.5
**CD8**	BV510	BioLegend	100751	1.25
**CD19**	BUV661	BD	612971	2.5
**CD11b**	BUV395	BD	563553	2.5
**CD11c**	PE-Dazzle594	BioLegend	117347	2.5
**CD103**	BV421	BD	562771	2.5
**CD24**	APC-Cy7	BioLegend	101849	5
**SiglecF**	BV605	BD	740388	2.5
**Ly6G**	PE	BioLegend	127607	2.5
**CD3**	AF700	BioLegend	100215	10
**CD64**	BV711	BioLegend	139311	10
**I-A/I-E**	Pe-Cy7	BioLegend	107629	0.3

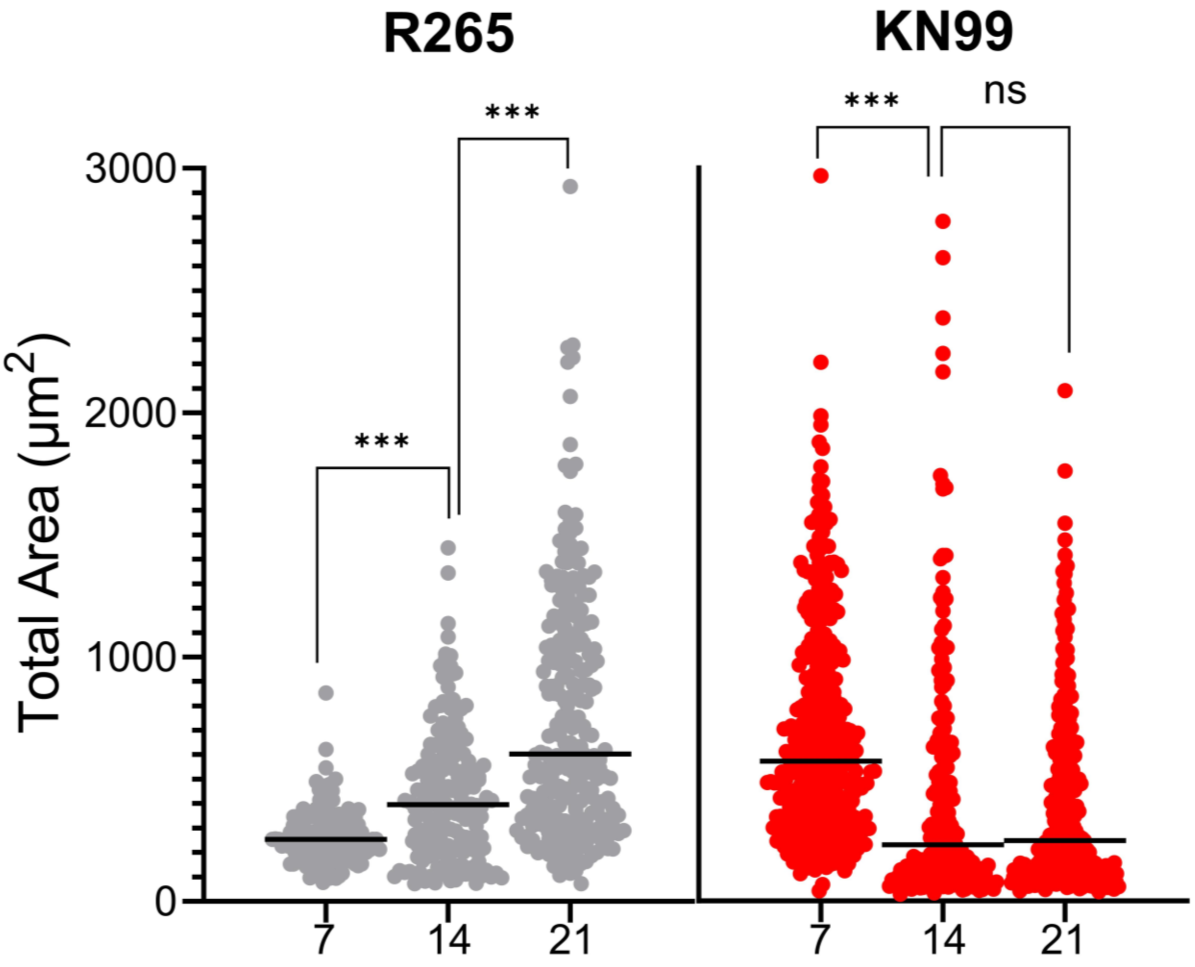

Figure S1: Total cell + capsule size over time. The total cell diameter was measured to calculate the total cell area as described in **Figure 1**. Horizontal bars represent the median. Each dot represents a single cell. X-axis represents day post infection. Significance was determined by one-way ANOVA with Sidak's multiple comparisons test. *Not significant (ns) at $P>0.05$; *** significant at $P\leq 0.001$.*

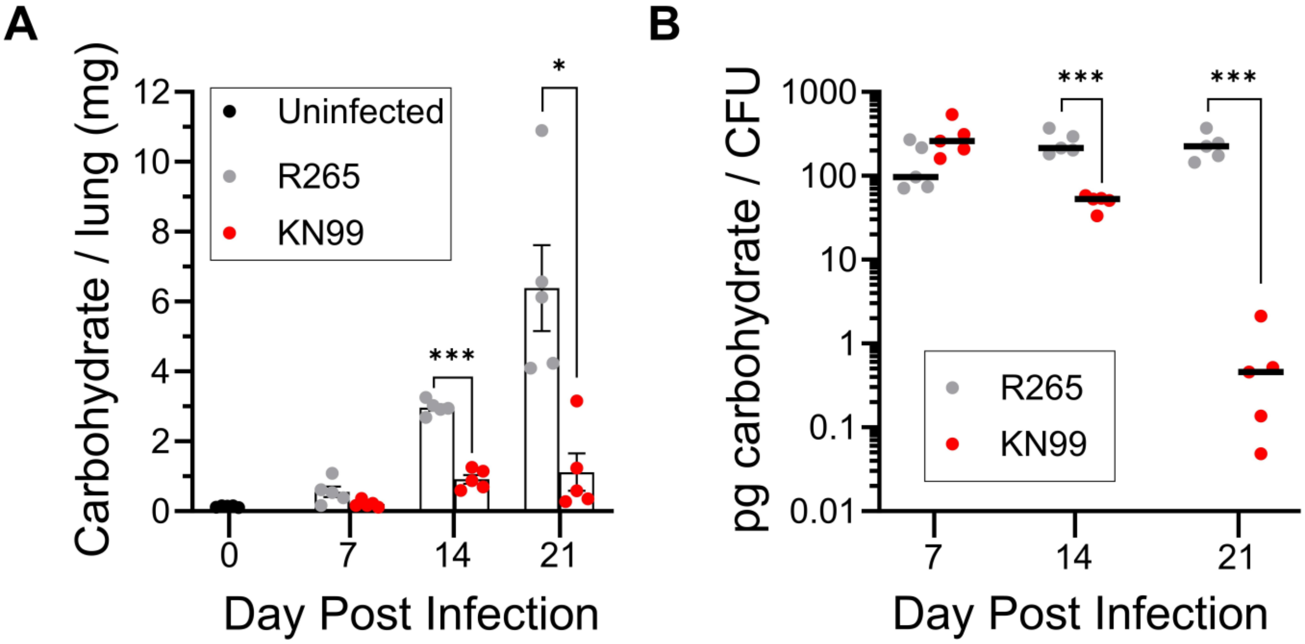

Figure S2: Carbohydrate content in the supernatants of lung homogenates from infected mice, determined by phenol-sulfuric assay. **(A)** Total carbohydrate, black dots represent uninfected mice. **(B)** Carbohydrate normalized to fungal burden in the lungs. Each group had 5 mice. Significance was determined by unpaired, two-tailed T-tests after applying the Bonferroni correction for multiple comparisons. *Not significant at P>0.05 (non-significant comparisons are unmarked); * significant at P≤0.05; *** significant at P≤0.001.*

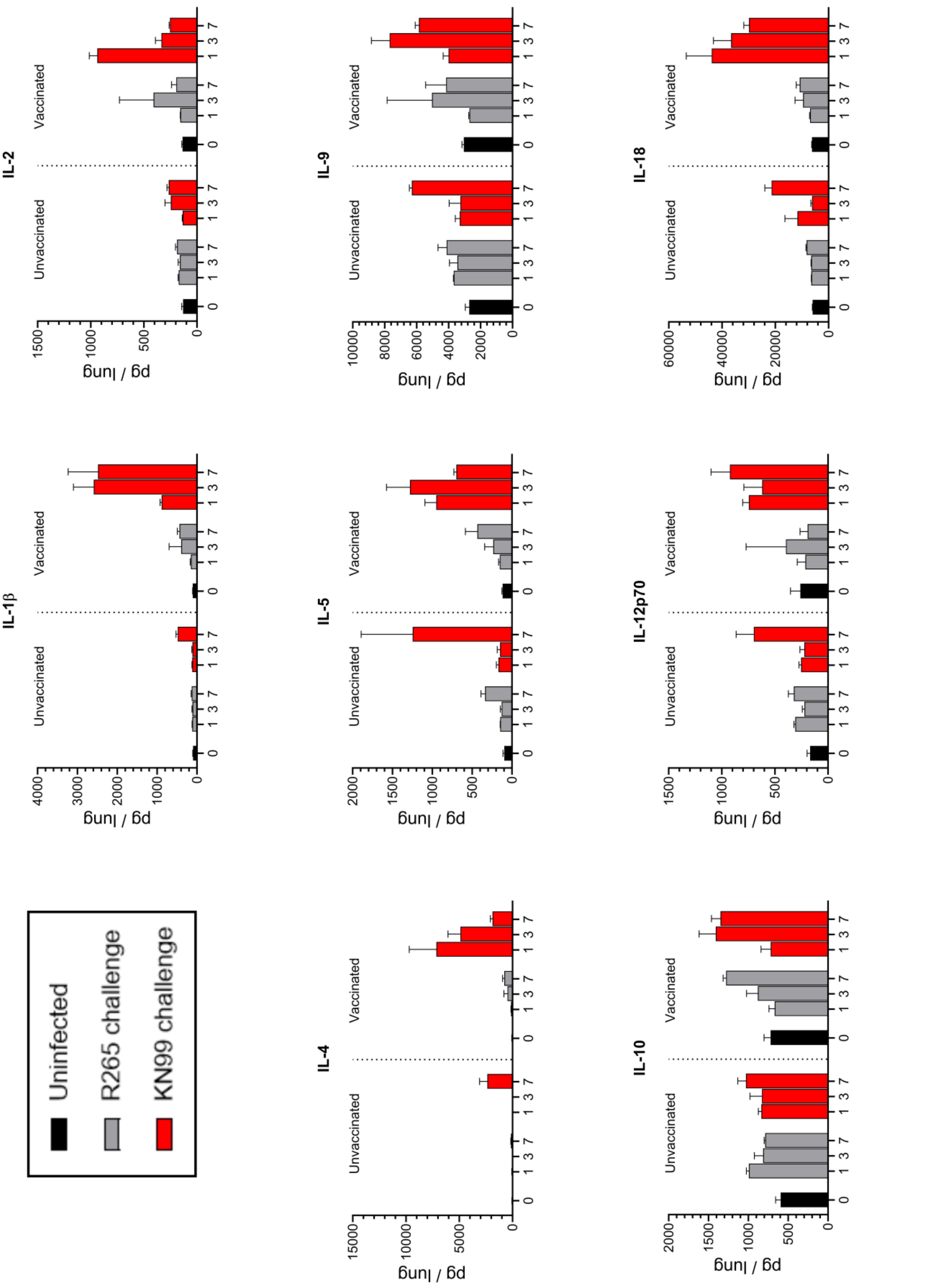

Uninfected
R265 challenge
KN99 challenge
IL-1β
IL-2
IL-4
IL-5
IL-9
IL-10
IL-12p70
IL-18
Unvaccinated
Vaccinated
pg / lung

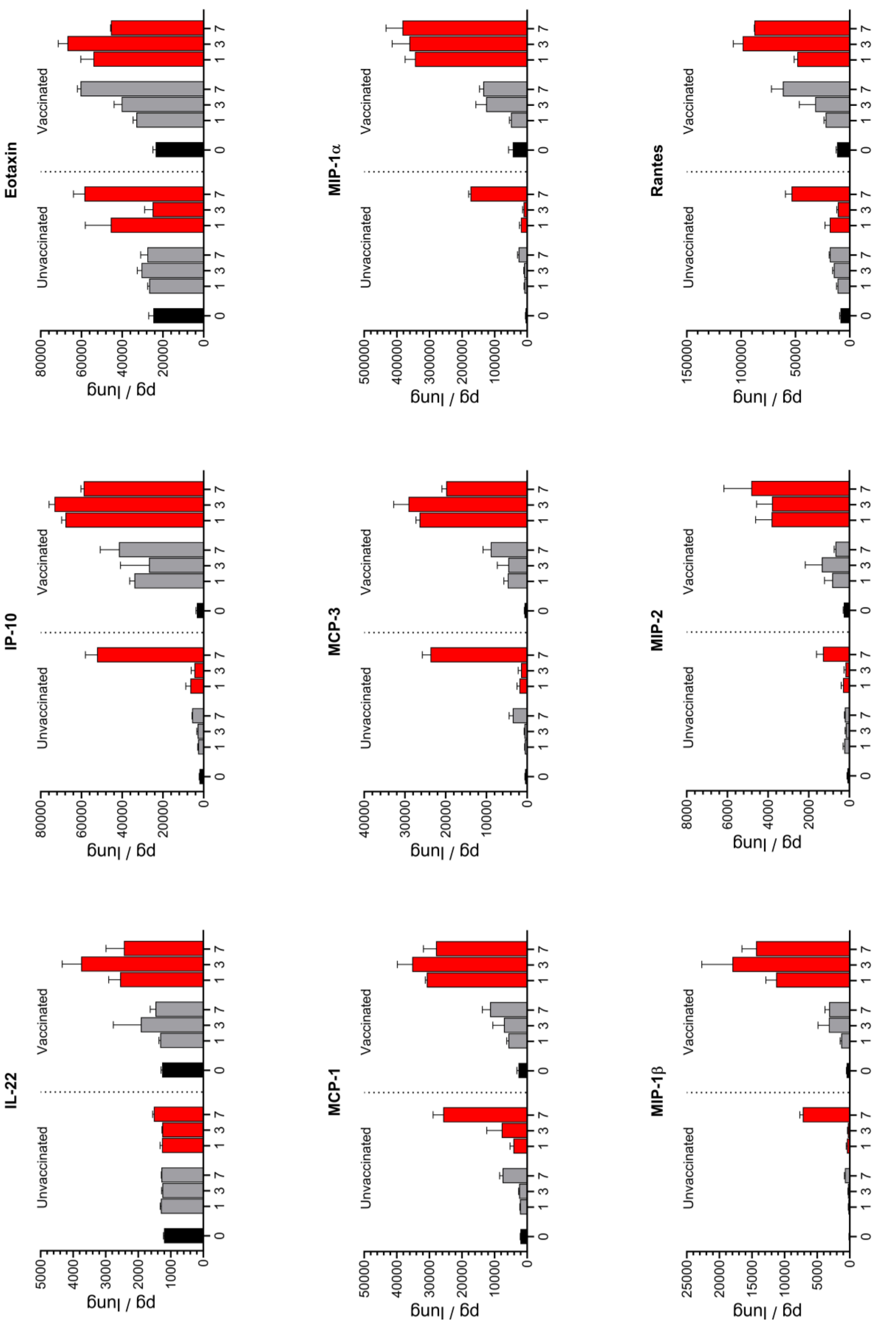
IL-22
IP-10
Eotaxin
MCP-1
MCP-3
MIP-1α
MIP-1β
MIP-2
Rantes
Unvaccinated
Vaccinated
pg / lung
0
1
3
7

Figure S3: Additional cytokines and chemokines quantified by Luminex 26-plex. BALB/c mice were vaccinated and challenged, and at the indicated time points, the mice were euthanized, and their lungs and serum (**Figure S4**) were processed for cytokine multiplex analysis. Data are displayed as in **Figure 2**, with the Y-axis representing either the total cytokine per lung, and the X-axis indicates the day post infection on which samples were collected. Unvaccinated groups are on the left, and vaccinated groups are on the right for uninfected (black), R265-infected (grey), and KN99-infected (red) mice. For each group/timepoint, *n*=3 mice. Bars represent the mean plus the standard error of the mean. Of the 26 analytes, three had no statistical difference between groups and are not shown (IL-13, IL-23, and IL-27).

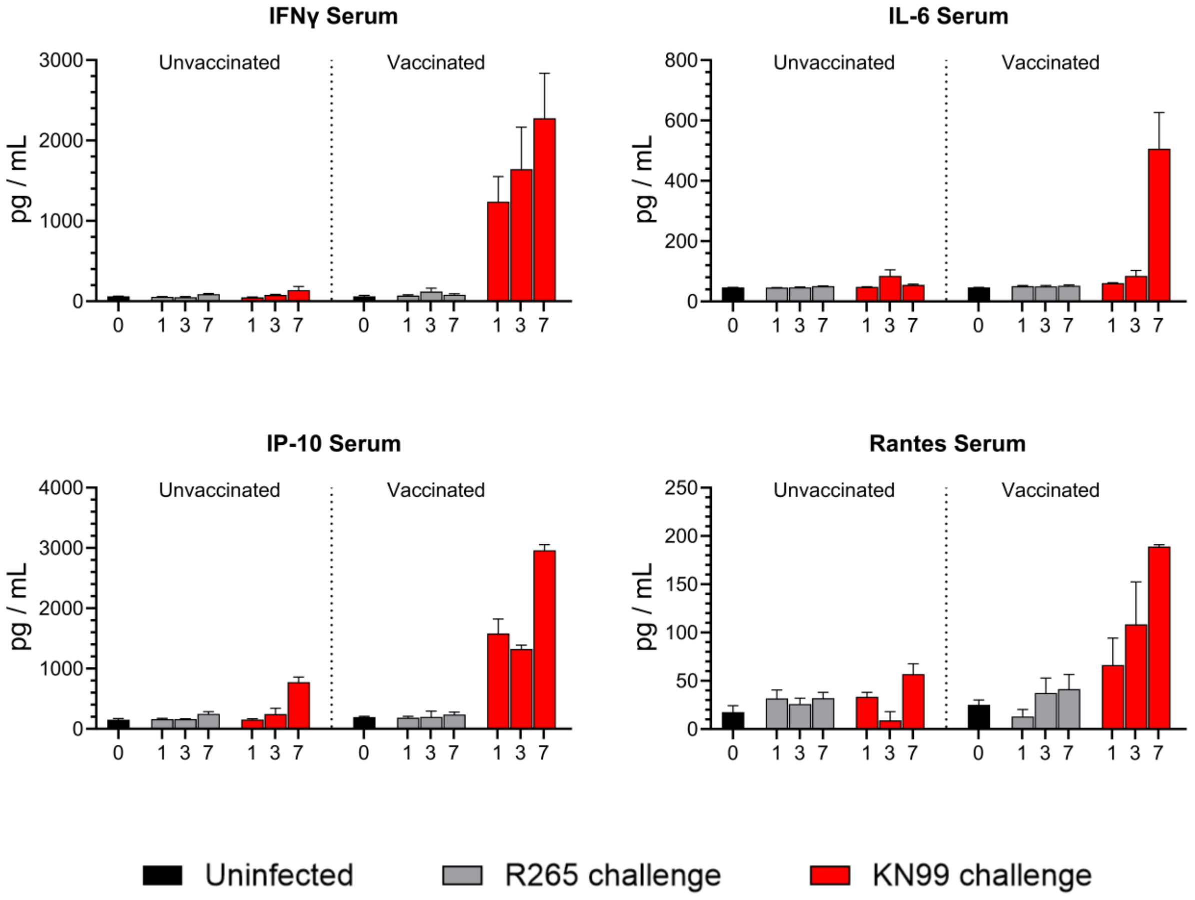

Figure S4: Serum cytokines quantified by Luminex 26-plex. BALB/c mice were vaccinated, challenged, and samples were processed as described in **Figure S3**. Data is displayed as in **Figure S3**, but the Y-axis represents cytokine levels in pg per mL of serum.

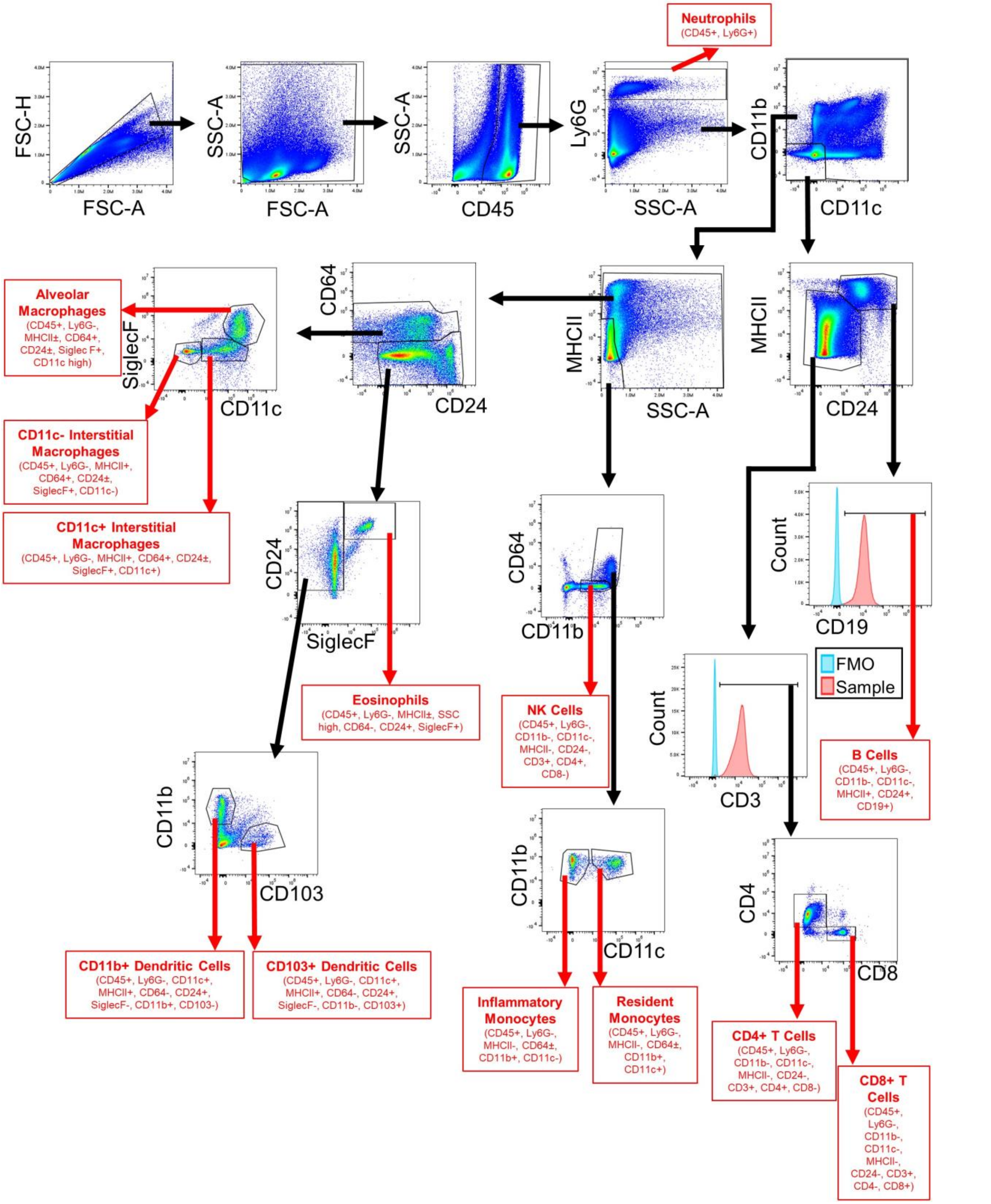

Figure S5: Gating Scheme for Flow Cytometry. Unmixed spectral data was analyzed using FlowJo. FMOs were used to determine gates. Gating strategies were adapted from "*A Protocol for the Comprehensive Flow Cytometric Analysis of Immune Cells in Normal and Inflamed Murine Non-Lymphoid Tissues*," (1).

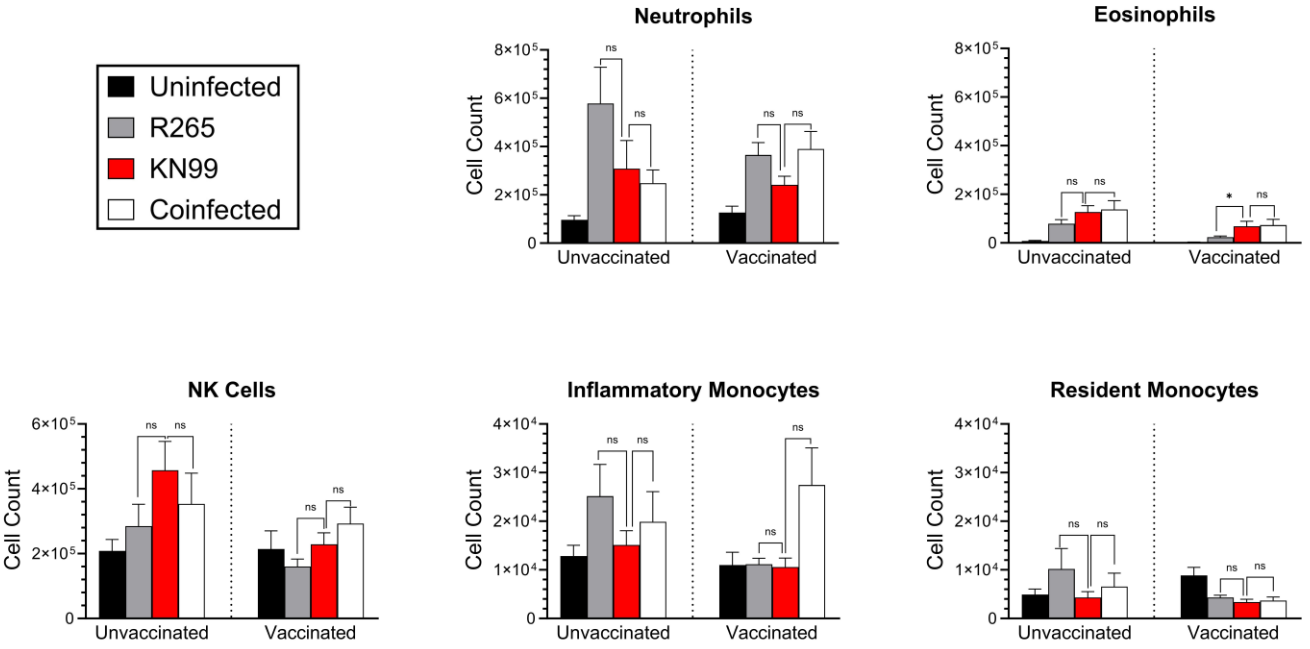

Figure S6: Additional immune cell phenotyping of lung cells in coinfected BALB/c mice. Samples for flow cytometry were collected and processed as described in **Figure 7**. Unmixed spectral data were analyzed according to the gating strategy outlined in **Figure S5**. Total leukocytes were quantified and then further gated to identify specific cell populations. Bars represent the mean plus the standard error of the mean. *n*=10 to14 mice per group. Each group is the cumulative result of 3 separate experiments, with the exception of the vaccinated, unchallenged group being only two separate experiments. Statistical significance was determined by unpaired, two-tailed t tests. *Not significant (ns) at P>0.05.*

References

1. Yu YR, O'Koren EG, Hotten DF, Kan MJ, Kopin D, Nelson ER, et al. A protocol for the comprehensive flow cytometric analysis of immune cells in normal and inflamed murine non-lymphoid tissues. PLoS One. 2016;11(3):e0150606.
